# Effects of astragaloside IV on glucocorticoid‐induced avascular necrosis of the femoral head via regulating Akt‐related pathways

**DOI:** 10.1111/cpr.13485

**Published:** 2023-04-26

**Authors:** Haojie Shan, Yiwei Lin, Fuli Yin, Chenhao Pan, Jianzhong Hou, Tianyi Wu, Wenyang Xia, Rongtai Zuo, Bojun Cao, Chaolai Jiang, Zubin Zhou, Xiaowei Yu

**Affiliations:** ^1^ Department of Orthopaedic Surgery Shanghai Sixth People's Hospital Affiliated to Shanghai Jiao Tong University School of Medicine Shanghai China; ^2^ Department of Orthopaedics & Traumatology, Li Ka Shing Faculty of Medicine The University of Hong Kong Hong Kong SAR China; ^3^ Department of General Surgery, Shanghai Fengxian Central Hospital Shanghai Jiao Tong University Affiliated Sixth People's Hospital South Campus Shanghai China; ^4^ Shanghai Key Laboratory of Orthopaedic Implants, Department of Orthopaedic Surgery, Shanghai Ninth People's Hospital Shanghai Jiao Tong University School of Medicine Shanghai China

## Abstract

We investigated the role of astragaloside IV (AS‐IV) in preventing glucocorticoid‐induced avascular necrosis of the femoral head (ANFH) and the underlying molecular mechanisms. Network pharmacology was used to predict the molecular targets of AS‐IV. Molecular dynamic simulations were performed to explore the binding mechanism and interaction mode between AS‐IV and Akt. Rat models of glucocorticoid‐induced ANFH with AS‐IV intervention were established, and osteogenesis, angiogenesis, apoptosis and oxidative stress were evaluated before and after blocking the PI3K/Akt pathway with LY294002. The effects of glucocorticoid and AS‐IV on bone marrow mesenchymal stem cells and human umbilical vein endothelial cells incubated with and without LY294002 were determined. Downregulated p‐Akt expression could be detected in the femoral heads of glucocorticoid‐induced ANFH patients and rats. AS‐IV increased trabecular bone integrity and vessel density of the femoral head in the model rats. AS‐IV increased Akt phosphorylation and upregulated osteogenesis‐, angiogenesis‐, apoptosis‐ and oxidative stress‐related proteins and mRNA and downregulated Bax, cleaved caspase‐3 and cytochrome c levels. AS‐IV promoted human umbilical vein endothelial cell migration, proliferation and tube formation ability; bone marrow mesenchymal stem cell proliferation; and osteogenic differentiation under glucocorticoid influence. AS‐IV inhibited apoptosis. LY294002 inhibited these effects. AS‐IV prevented glucocorticoid‐induced ANFH by promoting osteogenesis and angiogenesis via the Akt/Runx2 and Akt/HIF‐1α/VEGF pathways, respectively, and suppressing apoptosis and oxidative stress via the Akt/Bad/Bcl‐2 and Akt/Nrf2/HO‐1 pathways, respectively.

## Graphical abstract

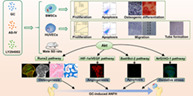

## 1 INTRODUCTION

Glucocorticoid (GC)‐induced avascular necrosis of the femoral head (ANFH) is a common adverse effect of GC administration in various inflammatory and autoimmune diseases, including systemic lupus erythematosus,^1^ acute lymphoblastic leukaemia,^2^ rheumatoid arthritis,^3^ nephrosis^4^ and severe acute respiratory syndrome.^5^ This accounts for the most non‐traumatic ANFH, representing 24.1% of all ANFH.^6^ GC‐induced ANFH causes pain and dysfunction of the hip joint, which seriously affects the quality of life and working ability of patients. Indeed, most patients with GC‐induced ANFH eventually undergo total hip arthroplasty. Therefore, the pathological mechanisms of GC‐induced ANFH should be investigated to determine novel therapeutic targets to disrupt ANFH progression.

The pathogenesis and molecular mechanisms contributing to the occurrence and development of GC‐induced ANFH remain unclear. However, abnormal osteogenic differentiation,^7^ vascular endothelial injury,^8^ cell apoptosis^9^ and oxidative stress^10^ triggered by GC have been suggested as underlying mechanisms. GC mainly acts on complex signalling pathways and molecules to induce ANFH, among which the protein kinase B (Akt) pathway plays a crucial role.^9^, ^11^, ^12^

Akt is the main downstream target of phosphoinositide 3‐kinase (PI3K) that consists of catalytic (p110) and regulatory (p85) subunits. After the interaction of PI3K with the cell surface receptors, p110 is activated to produce the intracellular secondary messenger phosphatidylinositol‐3,4,5⁃trisphosphate, which transfers Akt to the cell membrane and activates it. Additionally, Akt further activates the downstream target genes, glycogen synthase kinase‐3β, mammalian target of rapamycin, nuclear factor kappa B, Bcl‐xL/Bcl‐2‐associated death promoter (Bad), nuclear factor erythroid 2‐related factor 2 (Nrf2) and hypoxia‐inducible factor‐1α (HIF‐1α), regulating cell proliferation, differentiation and apoptosis.^13^ Akt acts on runt‐related transcription factor 2 (Runx2) to regulate osteogenesis^14^ and on the HIF‐1α/vascular endothelial growth factor (VEGF) pathway to regulate angiogenesis^15^; Nrf2/heme oxygenase‐1 (HO‐1) pathway to regulate oxidative stress^16^; and Bad/B‐cell lymphoma 2 (Bcl‐2) pathway to regulate apoptosis.^9^

Therefore, targeted regulation of Akt and its downstream related pathways to promote osteogenesis or angiogenesis and inhibit apoptosis or oxidative stress might offer effective means for the early prevention of GC‐induced ANFH. Among the several methods applied to prevent GC‐induced ANFH, monomers extracted from Chinese herbs have aroused widespread attention and may provide profound therapeutic benefits.^12^, ^17^ Astragaloside IV (AS‐IV; 3‐O‐β‐d‐xylopyranosyl‐6‐O‐β‐d‐glucopyranosyl‐cycloastragenol; Figure S1A) extracted from *Astragalus membranaceus* (Fisch.) Bunge has been widely applied to treat ischemic diseases in traditional Chinese medicine.^18^, ^19^ AS‐IV exerts various pharmacological effects, including proangiogenic,^20^, ^21^ pro‐osteogenic,^22^ antiapoptotic^23^, ^24^ and anti‐oxidative effects.^23^, ^25^ AS‐IV can act on the Akt signalling pathway to exert biological effects.^20^, ^26^, ^27^ However, the role and potential action mechanisms of AS‐IV in the prevention of GC‐induced ANFH have not been systematically addressed, and the binding mechanism and interaction mode between AS‐IV and Akt are unknown. We speculated that AS‐IV could prevent the progression of GC‐induced ANFH through different mechanisms via Akt‐mediated pathways.

## 2 MATERIALS AND METHODS

### 2.1 Network pharmacologic analysis

The chemical structure of AS‐IV was obtained from the PubChem database (http://pubchem.ncbi.nlm.nih.gov/). The targets were predicted using the PharmMapper database (http://www.lilab-ecust.cn/pharmmapper/) after inputting the SDF file of AS‐IV. Related disease targets of GC‐induced ANFH were obtained from the GeneCards database (https://www.genecards.org/) and Online Mendelian Inheritance in Man (OMIM, https://www.omim.org/). The intersecting targets were input into the STRING database (https://cn.string-db.org/) and the core proteins were topologically analysed using Cytoscape 3.7.2. Common targets were imported into DAVID Bioinformatics Resources 6.8 (https://david.ncifcrf.gov/home.jsp) for Gene Ontology (GO) and Kyoto Encyclopedia of Genes and Genomes (KEGG) pathway enrichment analyses.

### 2.2 Molecular docking

The three‐dimensional structures of AS‐IV and Akt were obtained from the PubChem database (http://pubchem.ncbi.nlm.nih.gov/) and the PDB website (https://www.rcsb.org/), respectively, and were optimized using PyMOL software (version 2.0.1). A single gridbox with a centre at *x* = −23, *y* = 99, *z* = −66 Å and sizes of 42, 42 and 42 Å at respective edges encapsulated the active site. Molecular docking was conducted using AutoDock Vina software (version 1.1.2), according to published methods.^28^, ^29^

### 2.3 Molecular dynamic simulations

Molecular dynamic simulations were performed using the Amber18 software package.^30^ The Amber ff14SB forcefield parameter set was used to model all standard amino acid residues. Ligand parameters were prepared in the Antechamber module using the GAFF force field. All missing hydrogen atoms were added using the Leap module. Solvation was performed using TIP3P. The truncated octahedron cell was replicated in the whole space with periodic boundary conditions. First, energy minimization was performed, after which the entire systems were heated from 0 to 300 K in 50 ps, followed by 100 ns molecular dynamic simulation with a time step of 2 fs. For various quantitative analyses, various tools from *cpptraj* were used. The binding free energy of protein–ligand was evaluated using the Molecular Mechanics/Generalized Born Surface Area (MM/GBSA) approach.^31^ A minimum distance of <0.4 nm was defined as a contact. A hydrogen bond was counted if the distance between the donor and acceptor atoms was <3.5 Å with a minimum donor–hydrogen–acceptor angle of 120°.

### 2.4 Patient samples

Fifteen GC‐induced ANFH patients (Table S1) who underwent hip arthroplasty in our department were enrolled from 2021 to 2022. These patients were diagnosed as Association Research Circulation Osseous classification stages II or III in accordance with the judgement of magnetic resonance imaging (MRI). The diagnosis was also verified using preoperative X‐ray and computed tomography (CT). We evaluated another 15 patients with fresh femoral neck fractures who underwent hip arthroplasty (excluding other types of bone and joint diseases) as the normal control group. The femoral heads were obtained during the operation. The characteristic information of patients involved in this study is provided in Table S1. The protocol was approved by the Ethics Committee of Shanghai Sixth People's Hospital affiliated with Shanghai Jiao Tong University School of Medicine, and consent forms were signed by the participants.

### 2.5 Animals

This study was approved by The Institutional Animal Care and Use Committee of Shanghai Jiao Tong University Affiliated Sixth People's Hospital (approval number: DWLL2021‐0928). Eight‐week‐old male Sprague–Dawley rats (*n* = 80) were randomly and evenly assigned to the control group and the following model groups: methylprednisolone (MPS), MPS + AS‐IV and MPS + AS‐IV + LY294002. Figure 2A illustrates the establishment of the animal model.^9^, ^32^, ^33^, ^34^ Briefly, MPS (20 mg/kg/day; Pfizer, Puurs, Belgium) dissolved in normal saline was injected intramuscularly on 3 consecutive days weekly for 3 weeks to induce ANFH.^9^, ^32^ AS‐IV (20 mg/kg/day) suspended in 1% carboxymethyl cellulose solution was administered to the rats via oral gavage from the first MPS injection and continued for 6 weeks.^33^ The rats in the control and MPS groups received equal volumes of carboxymethyl cellulose solution. LY294002 (0.3 mg/kg/day) was administered intraperitoneally for 6 weeks.^34^

### 2.6 Sequential fluorescent labelling

To detect new bone formation, tetracycline (25 mg/kg), alizarin red (30 mg/kg) and calcein (20 mg/kg)—dissolved in normal saline—were injected intraperitoneally at 0, 2 and 4 weeks after the first MPS injection (Figure 2A).^35^, ^36^

### 2.7 Micro‐CT scanning and analysis

After 6 weeks, the femoral heads were dissected from the rats, fixed in formalin for 72 h, and examined using a SkyScan‐1176 micro‐CT scanner (Bruker Corp., Billerica, NY) at the resolution of 9 μm/pixel. Three‐ and two‐dimensional images were acquired using CTvox v. 2.4.0.0 and DataViewer v. 1.4.4.0 (both from Bruker Corp.), respectively. Parameters of the regions of interest, including bone mineral density, bone volume/tissue volume, trabecular thickness, trabecular number, trabecular separation and connectivity density, were analysed using CTAn v.1.13.2.1 (Bruker Corp.).

### 2.8 Angiography

The rats were anaesthetized, the proximal abdominal aorta was ligated and the inferior vena cava was cut off. Blood vessels were flushed with heparinized saline through an indwelling needle inserted into the abdominal aorta until complete removal of the blood, and the tissues and vessels were fixed in 4% paraformaldehyde. Thereafter, we injected Microfil (MV‐122; Flow Tech, Inc., Carver, MA) through an indwelling needle until the outflow of Microfil from the inferior vena cava was constant. The rats were kept at 4°C overnight, and then the femoral heads were removed, fixed in 4% paraformaldehyde and decalcified using 10% EDTA. The samples were examined using the SkyScan‐1176 micro‐CT scanner, and three‐dimensional images of the vasculature were generated using CTvox v. 2.4.0.0. Vessel volume, volume percentage, diameter and number were calculated using CTAn v.1.13.2.1.

### 2.9 Haematoxylin and eosin and Masson staining

The decalcified femoral heads of patients and rats were paraffin‐embedded and cut into 4‐μm‐thick sections using a microtome (Leica Microsystems, Wetzlar, Germany). The sections were stained with haematoxylin and eosin and a Masson trichrome staining kit (Solarbio Co., Ltd., Beijing, China). Images were acquired using an ECLIPSE80i microscope (Nikon, Tokyo, Japan), and the proportion of empty lacunae was determined.

### 2.10 Immunohistochemical and immunofluorescence staining

The paraffin‐embedded sections for immunohistochemical staining were deparaffinized and incubated with 3% hydrogen peroxide, followed by antigen retrieval. Thereafter, the sections were incubated with 1% bovine serum albumin; afterwards, they were incubated with anti‐VEGFA, anti‐von Willebrand factor (vWF), anti‐CD31 and anti‐Bcl‐2 as primary antibodies and horseradish peroxidase‐conjugated secondary antibodies. The sections were stained with 3,3′‐diaminobenzidine, counterstained with haematoxylin and examined using the ECLIPSE80i microscope. The paraffin‐embedded sections of patients and rats for immunofluorescence staining were deparaffinized, and antigens were retrieved. Nonspecific protein binding was blocked with 1% bovine serum albumin. The sections were incubated with anti‐p‐Akt, anti‐collagen I, anti‐HIF‐1α, anti‐HO‐1, anti‐nicotinamide adenine dinucleotide phosphate quinine oxidoreductase‐1 (NQO‐1), anti‐Nrf2 and anti‐Runx2 antibodies for 2 h, and then with secondary antibodies for 1 h. Cell nuclei were visualized using 4′,6‐diamidino‐2‐phenylindole. Images were captured using an IX 70 fluorescence microscope (Olympus, Tokyo, Japan).

### 2.11 TdT‐mediated dUTP nick end labelling assay and Ki67 immunostaining

After decalcification, DNA strand breaks were detected using a TdT‐mediated dUTP nick end labelling staining kit (Roche, Nutley, NJ). Cell proliferation was detected using the Ki67 antibody (Abcam, Cambridge, UK), and the nuclei were stained with 4′,6‐diamidino‐2‐phenylindole. Images were captured using the IX 70 fluorescence microscope (Olympus).

### 2.12 Confocal microscopy

The samples fixed in 4% paraformaldehyde were dehydrated, embedded in polymethylmethacrylate and cut into 150‐μm‐thick sections using a saw microtome. Images of fluorescently labelled specimens were acquired using a laser scanning confocal microscope (Leica Microsystems) with excitation/emission wavelengths of 543/617 nm for alizarin red, 488/517 nm for calcein and 405/580 nm for tetracycline.^35^

### 2.13 Western blotting

The femoral heads from patients and rats were dissected, snap‐frozen in liquid nitrogen, powdered and homogenized. The total proteins in the femoral head tissues were isolated using the Minute Total Protein Extraction Kits for Bone Tissues (Invent Biotech, Beijing, China). The expression of p‐Akt, Akt, collagen I, osteopontin (OPN), Runx2, HIF‐1α, VEGFA, Bad, p‐Bad, Bcl‐2, cleaved caspase‐3 (CC3), cytochrome c, Bcl‐2‐associated X protein (Bax), NQO‐1, Nrf2, HO‐1 and β‐actin was analysed as previously described.^37^

### 2.14 Quantitative real‐time PCR

Primers were designed and synthesized by Tsingke Biotechnology Co., Ltd. (Shanghai, China). Primer sequences are listed in Table S2. Total RNA was isolated from the femoral head tissues using TRIzol (Invitrogen, Waltham, MA) following the manufacturer's protocol. First‐strand cDNA synthesis was performed using a RevertAid First Strand cDNA Synthesis Kit (Thermo Scientific, Waltham, MA). qRT‐PCR was performed in an ABI 7300 Real‐Time PCR system (Applied Biosystems, CA) using FastStart Universal SYBR Green Master kit (ROX; Roche, Toronto, Canada). mRNA levels were calculated using the 2^−ΔΔCt^ method and normalized to GAPDH expression.

### 2.15 Cell culture

Human bone marrow mesenchymal stem cells (BMSCs; Cell Bank of the Chinese Academy of Sciences, Shanghai, China) were maintained in α‐MEM (HyClone, Logan, UT) containing 1% penicillin/streptomycin (HyClone) and 10% foetal bovine serum (Gibco Laboratories, Gaithersburg, MD). Human umbilical vein endothelial cells (HUVECs; ScienCell Research Laboratories Inc., Carlsbad, CA) were maintained in endothelial cell medium (ScienCell) containing 1% penicillin/streptomycin solution, 1% endothelial cell growth supplement and 5% foetal bovine serum. The cells were used between the third and sixth passages (p3–p6) and divided into six groups for the cell counting kit‐8 (CCK‐8) assay: (1) control; (2) dexamethasone (DEX; 10 μM; Solarbio Co., Ltd.; concentration was selected based on previous studies^8^, ^9^); (3) DEX + 5 μM AS‐IV; (4) DEX + 20 μM AS‐IV; (5) DEX + 50 μM AS‐IV; and (6) DEX + 100 μM AS‐IV. For alizarin red staining; alkaline phosphatase (ALP) staining; and apoptosis, tube formation, scratch wound and Transwell migration assays, the cells were divided into control, DEX, DEX + AS‐IV and DEX + AS‐IV + LY294002 groups. DEX concentration was 10 μM for alizarin red staining; ALP staining; and tube formation, Transwell migration and scratch wound assays; and 100 μM for the apoptosis assay. AS‐IV and DEX were administered simultaneously. The concentrations of AS‐IV and LY294002 were 50 and 10 μM, respectively.^38^, ^39^

### 2.16 CCK‐8 assay

BMSC and HUVEC proliferation was evaluated using the respective CCK‐8 kits (Dojindo, Kumamoto, Japan). The cells (5 × 10^3^/well) were seeded in 96‐well plates and incubated with CCK‐8 for 2 h. The optical density of the samples was measured at 450 nm on days 0, 1, 3 and 5 using a microplate reader (BioTek, Winooski, VT).

### 2.17 Determination of cell apoptosis via flow cytometry

BMSC and HUVEC apoptosis was quantified using Annexin V‐FITC apoptosis kits (BD Biosciences, San Jose, CA). The cells (1 × 10^6^/mL) were resuspended in Annexin V binding buffer, and 1 × 10^5^ cells (100 μL) were then incubated with FITC Annexin V (5 μL) and propidium iodide (5 μL) in the dark for 15 min. Thereafter, the cell suspension was diluted to 250 μL with Annexin V binding buffer, and the cells were analysed by flow cytometry.

### 2.18 ALP and alizarin red staining

Osteogenic differentiation of BMSCs seeded in 24‐well plates was induced in osteogenic differentiation medium (Cyagen Biosciences, Santa Clara, CA) for 7 days, and ALP activity was evaluated using a BCIP/NBT ALP colour development kit (Solarbio Co., Ltd.). Calcium mineral deposits were detected by alizarin red staining (Cyagen) after induction for 21 days. Images were acquired using the IX 70 inverted microscope (Olympus), and then the ratio of stained to total areas was calculated.

### 2.19 Transwell migration assay

We seeded HUVECs (2 × 10^4^/well) in the upper chambers of 24‐well Transwell plates (Corning Inc., Corning, NY). A complete endothelial cell medium was added to the lower chambers as a chemoattractant. The membranes were stained with crystal violet (Beyotime) and examined 24 h later using an IX 70 microscope.

### 2.20 Scratch wound assay

After HUVECs seeded in six‐well plates reached 90%–100% confluence, the complete medium was replaced with a serum‐free medium. The cell layer in each well was scratched using a 200‐μL micropipette tip. Digital images were captured 0, 12 and 24 h later using an IX 70 microscope, and cell migration was quantified using ImageJ.

### 2.21 Tube formation assay

Dissolved Matrigel (Corning Inc.) was added (200 μL/well) into 24‐well plates on ice and then solidified by incubation for 30 min at 37°C. Thereafter, HUVECs (1.2 × 10^5^/well) were resuspended in endothelial cell medium and seeded in the 24‐well plates. Images were acquired 6 h later using an IX 70 microscope (Olympus). The total mesh area, total length and number of nodes were measured using ImageJ.

### 2.22 Statistical analysis

All experiments were repeated at least thrice. CCK‐8 assay data were evaluated using the two‐way analysis of variance, while other data were analysed using two‐tailed unpaired *t*‐tests using GraphPad Prism 8.0. Data are shown as mean ± standard deviation. Values with *p* < 0.05 were considered significantly different.

## 3 RESULTS

### 3.1 Akt is a bona fide target of AS‐IV in preventing GC‐induced ANFH

**FIGURE 1:**
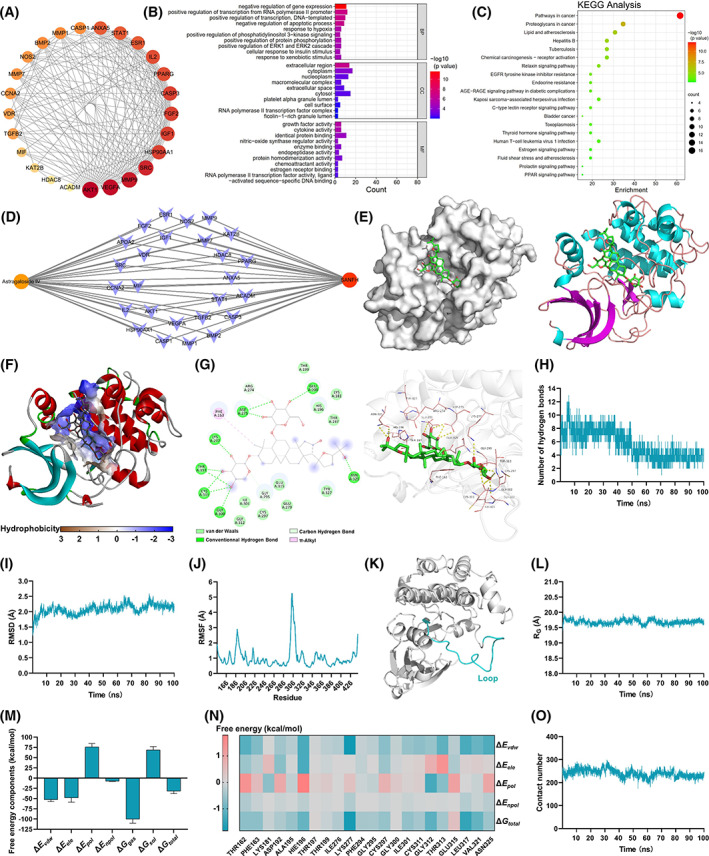
Akt is a bona fide target of AS‐IV in preventing GC‐induced ANFH. (A) PPI analysis. The heavier the colour, the more important the gene. (B) GO enrichment analysis. (C) KEGG pathway analysis. (D) Network of ‘AS‐IV‐Disease‐Target’. (E) Representative images of autodocking for Akt and AS‐IV. (F) Hydrophobicity of binding site. (G) Interaction maps for the protein–ligand complex. (H) Time evolution of the number of hydrogen bonds. (I) Time evolution of RMSD. (J) RMSF. (K) High flexibility region dominated by loop (blue). (L) Time evolution of *R*_G_. (M) Contributions of the binding free energy components. (N) Residue‐specific protein–ligand interaction energy and its decomposition. (O) Time distribution function for the contact number between Akt and AS‐IV.

Network pharmacology was used to predict the molecular targets of AS‐IV. As shown in Figure S1B, the number of the corresponding targets of AS‐IV was 377, and the number of the disease targets of GC‐induced ANFH was 359. Subsequently, the targets of AS‐IV were intersected with disease targets to give 26 genes, which were the targets of AS‐IV in repairing GC‐induced ANFH. Twenty‐six nodes and 160 edges were obtained in a protein–protein interaction (PPI) network (Figure 1A). The target with the maximum degree was Akt1. GO analysis was performed for enrichment analysis of biological processes (BP), cell components (CC) and molecular function (MF). For BP, AS‐IV treatment of GC‐induced ANFH mainly involved the response to ‘positive regulation of transcription from RNA polymerase II promoter’ and ‘negative regulation of gene expression’ (Figure 1B). For CC, genes mainly related to ‘extracellular region’ and ‘cytoplasm’ were remarkably enriched. For MF, the targets were closely related to ‘growth factor activity’ and ‘cytokine activity’. KEGG pathway enrichment analysis showed that AS‐IV‐associated genes were mainly linked to signalling cascade pathways, and Akt1 was the main gene in most KEGG signalling pathways (Figure 1C; Table S3). Subsequently, the network of ‘AS‐IV‐Disease‐Target’ was established (Figure 1D).

To assess the exact binding site of AS‐IV on Akt and the interactions at the atomic level, we performed molecular docking and molecular dynamics simulations. AS‐IV was bound in the binding pocket on the surface of Akt (affinity = −8.5 kcal/mol), and the two had a good shape match (Figure 1E). This binding site was highly hydrophilic (Figure 1F). Akt residues Asn325, Glu200, Asp275, Lys277, Thr313, Cys311 and Gly300 were involved in forming hydrogen bonds with the hydroxyl groups of AS‐IV, which was essential for stable binding (Figure 1G). Furthermore, AS‐IV formed π‐alkyl with Phe163.

The time evolution of the number of hydrogen bonds between AS‐IV and Akt is shown in Figure 1H. The number of hydrogen bonds ranged from 1 to 13 and finally stabilized at approximately 4. These stable hydrogen bonds were critical for ligand binding. The time evolution of root mean square displacement (RMSD) is shown in Figure 1I. The RMSD profile fluctuated smoothly, especially after 40 ns. The RMSD fluctuation stabilized at approximately 2 Å until the end of the simulations, indicating that AS‐IV bound to Akt without causing persistent, significant changes in protein conformation. Root mean square fluctuation (RMSF) is shown in Figure 1J. Amino acid regions in the 290–310 range of the protein core domain had greater flexibility than that of other regions, and this region was dominated by a loop (Figure 1K). Another structural parameter, radius of gyration (*R*_G_), was used to measure protein structure compactness. As shown in Figure 1L, *R*_G_ was relatively stable and tended to decrease slightly. We hypothesized that Akt structure tended to become compact, and AS‐IV bound tightly with Akt.

Contributions of the binding free energy components are shown in Figure 1M. Electrostatic (Δ*E*_ele_, −47.9 kcal/mol) and van der Waals (Δ*E*_vdw_, −52.5 kcal/mol) terms in the gas phase were found to be favourable for protein–ligand binding. The nonpolar part (Δ*E*_npol_, −7.6 kcal/mol) of the solvation‐free energy was favourable, but the polar part (Δ*E*_pol_, 76.1 kcal/mol) was highly unfavourable, resulting in an unfavourable total solvation‐free energy. The average interaction energy in the gas phase (Δ*G*_gas_, −100.4 kcal/mol) was the summation of Δ*E*_vdw_ and Δ*E*_ele_. The solvation‐free energy in the implicit aqueous phase (Δ*G*_sol_, 68.5 kcal/mol) was the summation of Δ*E*_pol_ and Δ*E*_npol_. Therefore, the total energy (Δ*G*_total_, −31.9 kcal/mol) was the summation of Δ*G*_gas_ and Δ*G*_sol_. The residue‐specific protein–ligand interaction energy and its decomposition are shown in Figure 1N and Table S4. Furthermore, the contact number between Akt and AS‐IV was approximately 250 and remained stable (Figure 1O), indicating that Akt and AS‐IV had enough contact points and the contact was stable, forming a stable complex.

After binding with AS‐IV, the hydroxyl radical of the Akt phosphorylation site (Ser473) side‐chain formed more intensive hydrogen bonding with the solvent; that is, the interaction with the solvent was stronger, especially after 80 ns (Figure S2A). Therefore, Akt Ser473 was more likely to be phosphorylated after binding with AS‐IV. In addition, after binding with AS‐IV, the hydrogen bonds between the hydroxyl radical of Akt Ser473 side‐chain and Akt weakened significantly, especially after 50 ns (Figure S2B). That is to say, the binding effect of Akt on the hydroxyl radical of Ser473 side‐chain was reduced, and phosphorylation occurred more easily. The combination of Akt and AS‐IV reduced the flexibility of residues around Ser473, and the conformation was relatively fixed, which made phosphorylation easier (Figure S2C). After binding with AS‐IV, the microenvironment of Akt phosphorylation site had a more open surface area (Figure S2D), which increased the exposure of the phosphorylation site and made phosphorylation easier.

### 3.2 Downregulated p‐Akt in GC‐induced ANFH patients and bone tissue‐protective effects of AS‐IV in GC‐induced ANFH rats

Cystic degeneration and bone loss occurred in patients with GC‐induced ANFH (Figure S3A). Immunofluorescence staining (Figure S3B) and western blotting (Figure S3C) showed that the p‐Akt level was considerably reduced in GC‐induced ANFH patients compared with that in the control patients.

**FIGURE 2:**
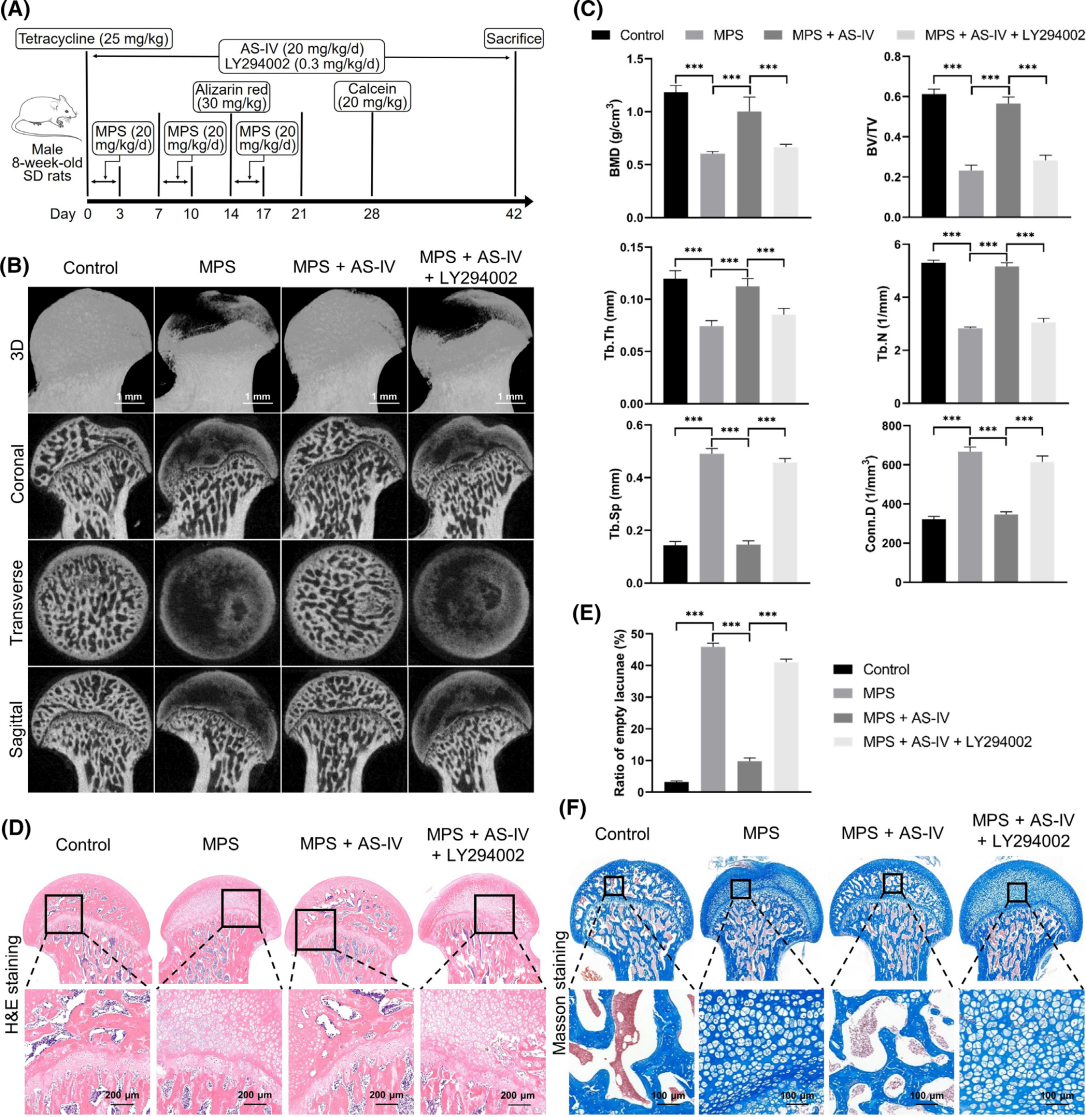
Bone tissue‐protective effects of AS‐IV in GC‐induced ANFH rats. (A) Workflow of the animal experiments. (B) Reconstructed three‐dimensional images of the transverse, coronal and sagittal sections of the femoral head (scale bar = 1 mm). (C) Quantitative analysis of the bone mineral density, bone volume/tissue volume, trabecular thickness, trabecular number, trabecular separation and connectivity density (*n* = 5). (D) Haematoxylin and eosin staining (scale bar = 200 μm). (E) Quantitative analysis of the ratios of empty lacunae stained with haematoxylin and eosin (*n* = 5). (F) Evaluation of collagen synthesis by Masson trichrome staining (scale bar = 100 μm). ***p < 0.001.

The bone structure of the femoral heads was evaluated by micro‐CT after 6 weeks of ANFH induction (Figure 2B). Cystic degeneration increased and a large trabecular bone area was lost in the subchondral trabeculae of the femoral heads in the model rats compared with those in the control rats. Conversely, the subchondral area of AS‐IV‐treated rats appeared intact and healthy. LY294002 increased bone loss and cystic degeneration. The bone mineral density, bone volume/tissue volume, trabecular number and trabecular thickness were considerably reduced, whereas trabecular separation and connectivity density were increased in the MPS group compared with those in the control group. However, AS‐IV considerably reversed these effects. Moreover, LY294002 reduced the bone mineral density, bone volume/tissue volume, trabecular number and trabecular thickness and increased trabecular separation and connectivity density again (Figure 2C).

Furthermore, haematoxylin and eosin staining (Figure 2D, E) indicated sparser or no subchondral trabeculae and a higher number of empty lacunae and pyknotic osteocytes in the MPS group than those in the control group. Contrarily, the number of empty lacunae and pyknotic osteocytes significantly decreased in the MPS + AS‐IV rats. However, the number of empty lacunae and pyknotic osteocytes increased in the MPS + AS‐IV + LY294002 group.

Masson trichrome staining showed considerably decreased collagen synthesis in the femoral heads of the MPS group, manifesting as reduction, thinning, sparse arrangement, breakdown or even dissolution of collagen fibres. Conversely, the collagen fibres thickened and increased in number with a good continuity in AS‐IV‐treated rats. However, LY294002 fragmented and dissolved collagen fibres again (Figure 2F).

### 3.3 AS‐IV rescued MPS‐induced inhibition of osteogenesis via the Akt/Runx2 pathway in vivo

**FIGURE 3:**
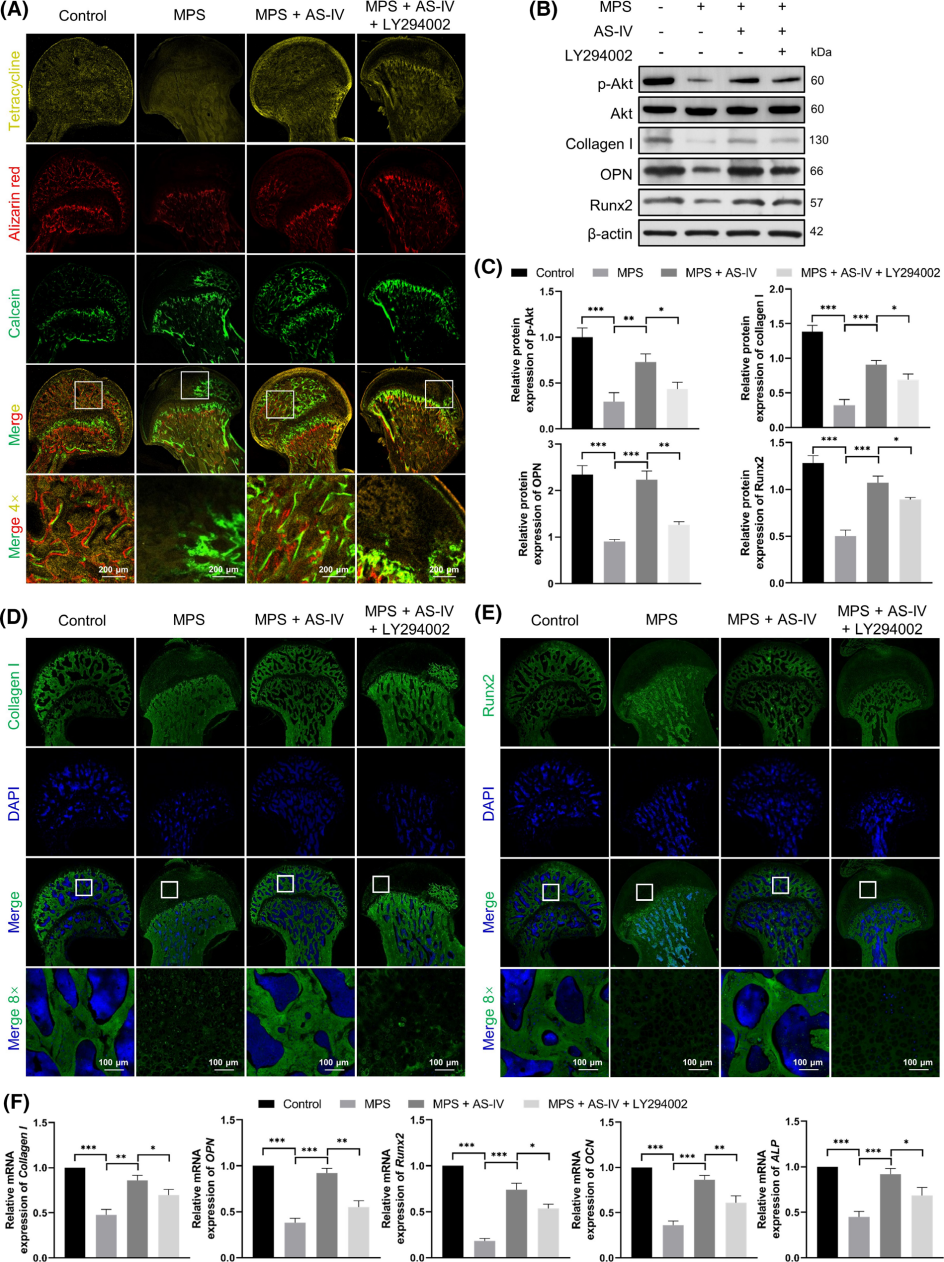
AS‐IV rescues MPS‐induced inhibition of osteogenesis via the Akt/Runx2 pathway in vivo. (A) Sequential fluorescent labelling to detect dynamic bone formation observed via confocal microscopy (scale bar = 200 μm). (B, C) Protein levels of p‐Akt, Akt, Runx2, collagen I and OPN assessed by western blotting (*n* = 3). (D, E) Immunofluorescent staining of Runx2 and collagen I (scale bar = 100 μm). (F) Relative mRNA levels of *Collagen I*, *OPN*, *Runx2*, *OCN* and *ALP* assessed by qRT‐PCR (*n* = 3). *p < 0.05, **p < 0.01 and ***p < 0.001.

Dynamic bone formation in the femoral head was monitored by sequential fluorescent labelling with tetracycline, alizarin red and calcein (Figure 3A). The staining intensity of the subchondral femoral head area with tetracycline (yellow), alizarin red (red) and calcein (green) was lower in the MPS group than that in the control group, indicating impaired bone homeostasis and decreased bone formation. AS‐IV apparently broadened the staining area of the femoral head. However, the distribution of the stained area was reduced in the MPS + AS‐IV + LY294002 group.

Next, we performed western blotting (Figure 3B,C), immunofluorescence staining (Figure 3D,E; Figure S4), and qRT‐PCR (Figure 3F). The Runx2, collagen I, OPN, osteocalcin (OCN), ALP and p‐Akt levels were considerably reduced in the MPS group compared with those in the control group. AS‐IV reversed these effects, whereas LY294002 decreased the Runx2, collagen I, OPN, OCN, ALP and p‐Akt levels.

### 3.4 AS‐IV rescued GC‐induced inhibition of BMSC proliferation and osteogenesis via the Akt pathway

**FIGURE 4:**
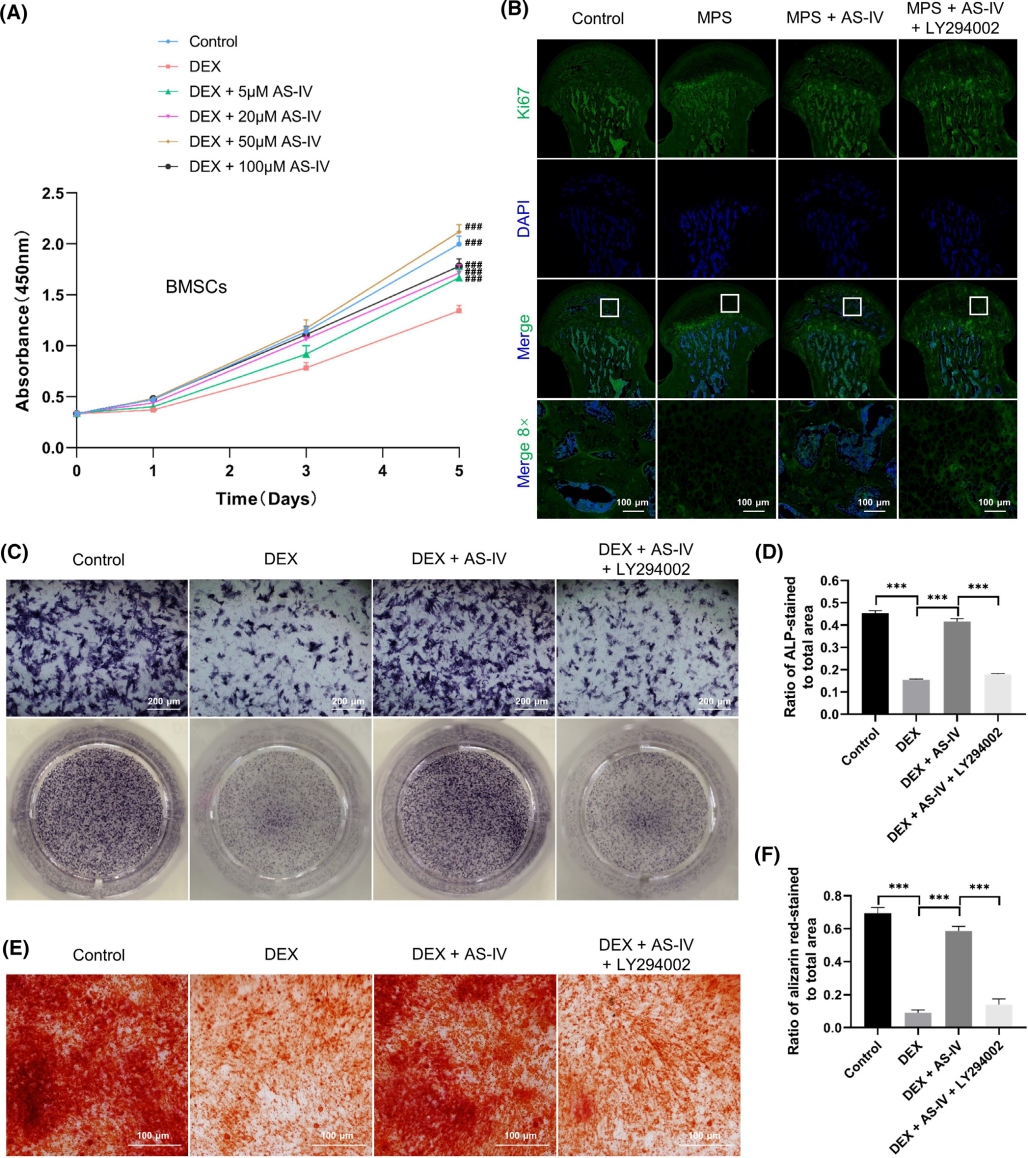
AS‐IV rescues GC‐induced inhibition of BMSC proliferation and osteogenesis via the Akt pathway. (A) Cell counting kit‐8 assay to evaluate BMSC proliferation under various conditions (*n* = 6). (B) Cell proliferation in the femoral heads evaluated by Ki67 immunostaining in vivo (scale bar = 100 μm). (C) Alkaline phosphatase (ALP) activity analysed by ALP staining (scale bar = 200 μm). (D) Ratio of ALP‐stained area to the total area (*n* = 3). (E) Calcium nodules stained with alizarin red (scale bar = 100 μm). (F) Ratio of alizarin red‐stained area to the total area (*n* = 3). ^###^ *p* < 0.001 versus the dexamethasone (DEX) group, ***p < 0.001.

DEX significantly reduced, whereas AS‐IV (50 μM) optimally increased BMSC proliferation (Figure 4A). We examined this effect of AS‐IV in the femoral heads of rats with MPS‐induced ANFH by Ki67 immunostaining (Figure 4B). Cell proliferation was lower in the MPS group than that in the control group. LY294002 suppressed AS‐IV‐induced cell proliferation.

The osteogenic activity of BMSCs after induction was evaluated via ALP and alizarin red staining. AS‐IV reversed the DEX‐induced osteogenic inhibition of BMSCs. However, calcium nodules and ALP activity were reduced in the DEX + AS‐IV + LY294002 group (Figure 4C–F).

### 3.5 AS‐IV rescued MPS‐induced inhibition of angiogenesis via the Akt/HIF‐1α/VEGF pathway in vivo

**FIGURE 5:**
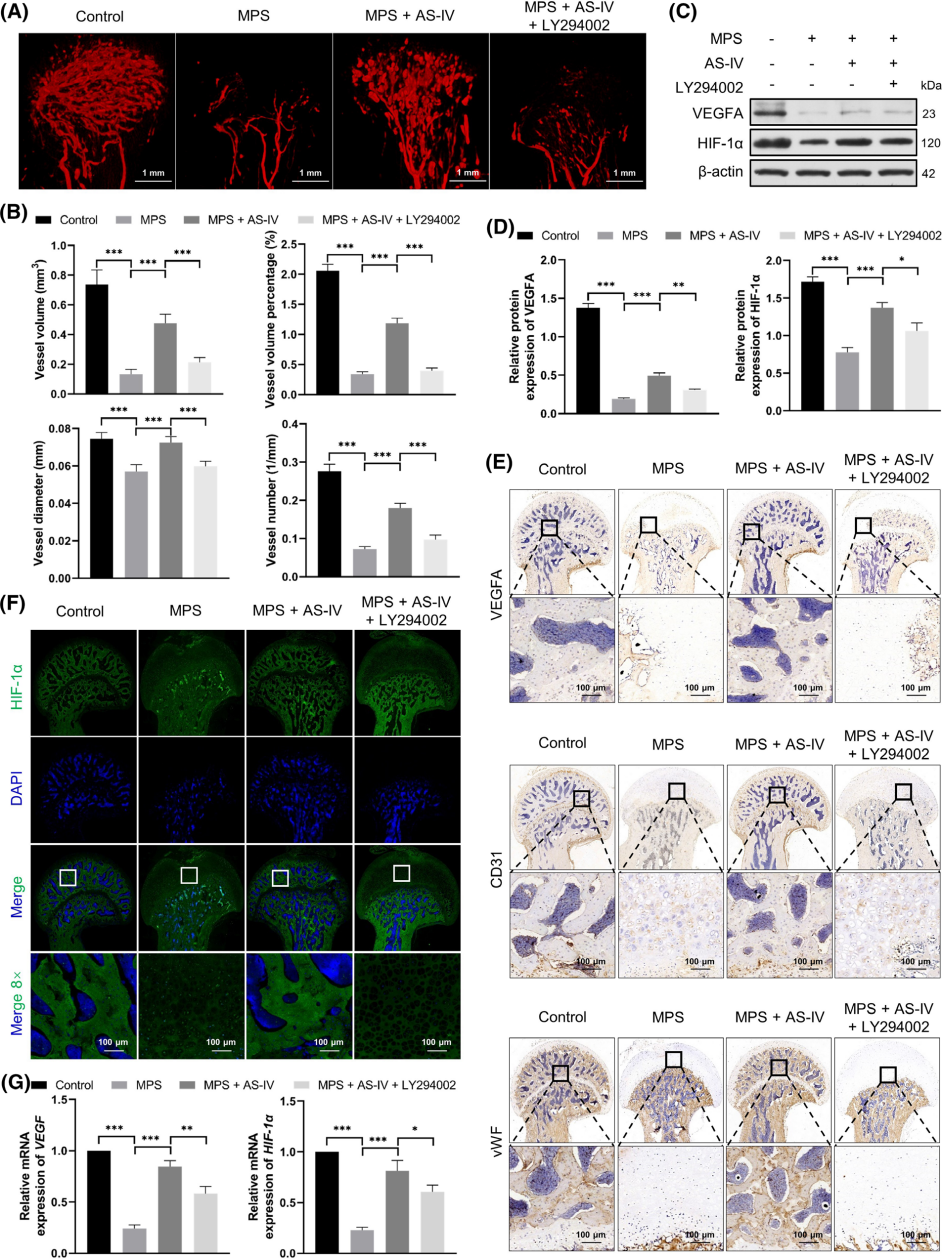
AS‐IV rescues MPS‐induced inhibition of angiogenesis via the Akt/HIF‐1α/VEGF pathway in vivo. (A) The blood supply in the femoral heads was detected by angiography (scale bar = 1 mm). (B) Quantitative analysis of the vessel volume, vessel volume percentage, vessel diameter and vessel number (*n* = 5). (C,D) The HIF‐1α and VEGFA protein levels examined by western blotting (n = 3). (E) Staining for VEGFA, CD31 and vWF proteins in samples by immunohistochemistry (scale bar = 100 μm). (*F*) Immunofluorescent staining of HIF‐1α in each experimental group (scale bar = 100 μm). (*G*) Relative mRNA levels of *HIF‐1α* and *VEGF* assessed by qRT‐PCR (*n* = 3). *p < 0.05, **p < 0.01 and ***p < 0.001.

As shown in Figure 5A, a higher number of vascular branches in the femoral head was observed in the MPS + AS‐IV group than those in the MPS group with an impaired vascular network. Notably, the vascular network was also impaired in the LY294002‐administered rats. MPS reduced the vessel volume, volume percentage, diameter and number in the MPS group compared with those in the control group, whereas AS‐IV reversed the effects, which were again reduced by LY294002 (Figure 5B).

The HIF‐1α, VEGFA, CD31 and vWF levels were examined by western blotting (Figure 5C,D), immunohistochemical staining (Figure 5E), immunofluorescence staining (Figure 5F) and qRT‐PCR (Figure 5G). The HIF‐1α, VEGFA, CD31 and vWF levels were lower in the MPS group than those in the control group. However, AS‐IV reversed these effects. LY294002 considerably reduced the HIF‐1α, VEGFA, CD31 and vWF levels.

### 3.6 AS‐IV rescued GC‐induced inhibition of HUVEC proliferation, migration and tube formation ability via the Akt pathway in vitro

**FIGURE 6:**
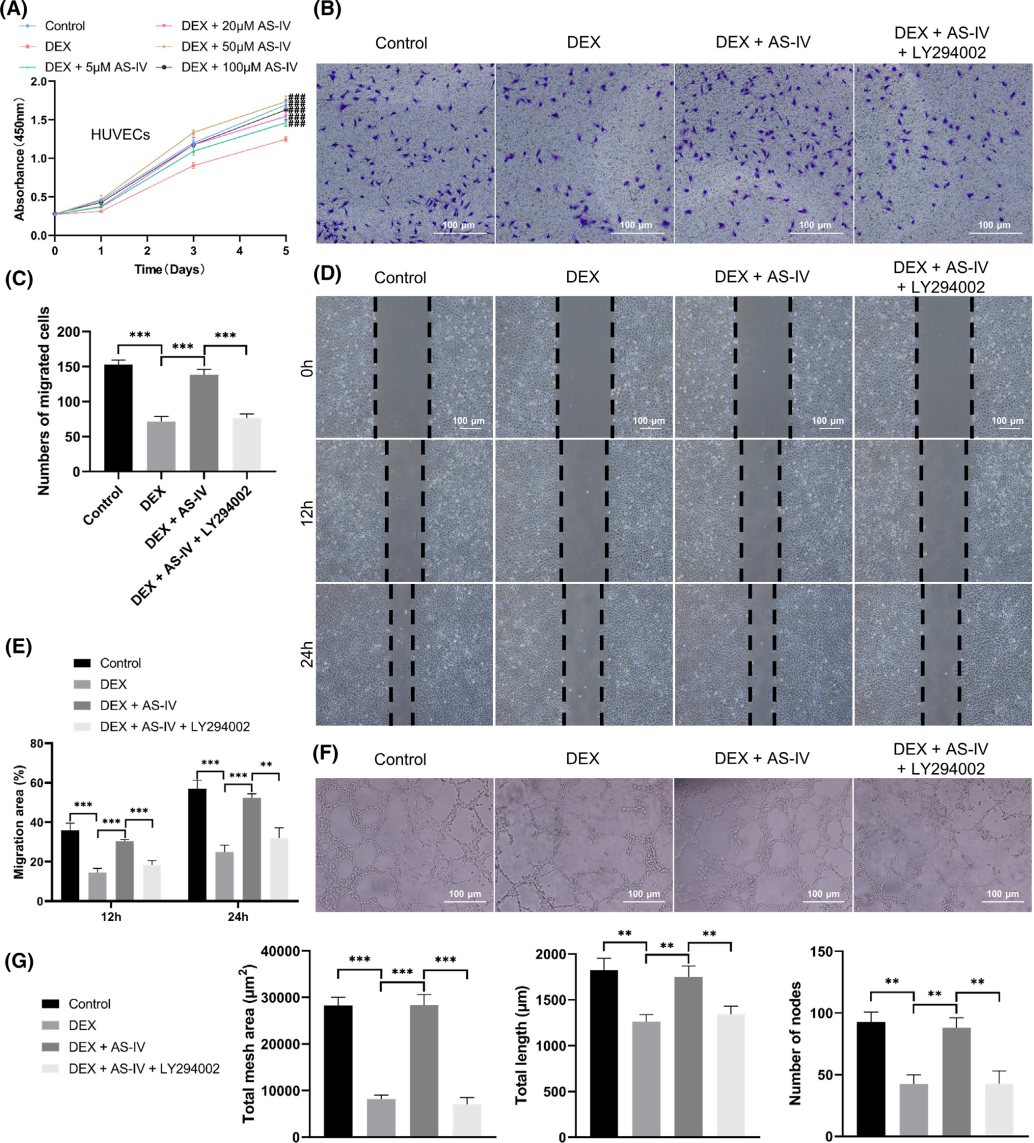
AS‐IV rescues GC‐induced inhibition of HUVEC proliferation, migration and tube formation in vitro. (A) HUVEC proliferation under various conditions assessed using the cell counting kit‐8 assay (*n* = 6). (B) Migration of HUVECs evaluated using the Transwell assay (scale bar = 100 μm). (C) Number of migrated cells in the Transwell assay (*n* = 3). (D) Migration of HUVECs evaluated using the scratch wound assay (scale bar = 100 μm). (E) Quantitation of HUVEC migration areas (*n* = 3). (F) Assessment of HUVEC tube formation ability (scale bar = 100 μm). (G) Quantitation of the total mesh area, total length and number of nodes in the tube formation assay (*n* = 3). ^###^ *p* < 0.001 versus the dexamethasone (DEX) group, **p < 0.01 and ***p < 0.001.

AS‐IV promoted HUVEC proliferation, which was attenuated by DEX, and AS‐IV (50 μM) exerted the most significant effects on cell proliferation (Figure 6A). The Transwell (Figure 6B,C) and scratch wound (Figure 6D,E) assays showed that DEX inhibited HUVEC migration compared with the control cells. However, AS‐IV significantly restored the number of migrated cells and the migration areas, which were reduced by LY294002. The tube formation assay showed that AS‐IV reversed DEX‐induced antiangiogenesis and increased the HUVEC loop formation ability (Figure 6F). AS‐IV increased the total mesh area, total length and number of nodes (Figure 6G). Conversely, the antiangiogenic effects were significant in the DEX + AS‐IV + LY294002 group when compared with those in the DEX + AS‐IV group (Figure 6F,G).

### 3.7 AS‐IV retarded GC‐induced apoptosis via the Akt/Bad/Bcl‐2 pathway in vivo and in vitro

**FIGURE 7:**
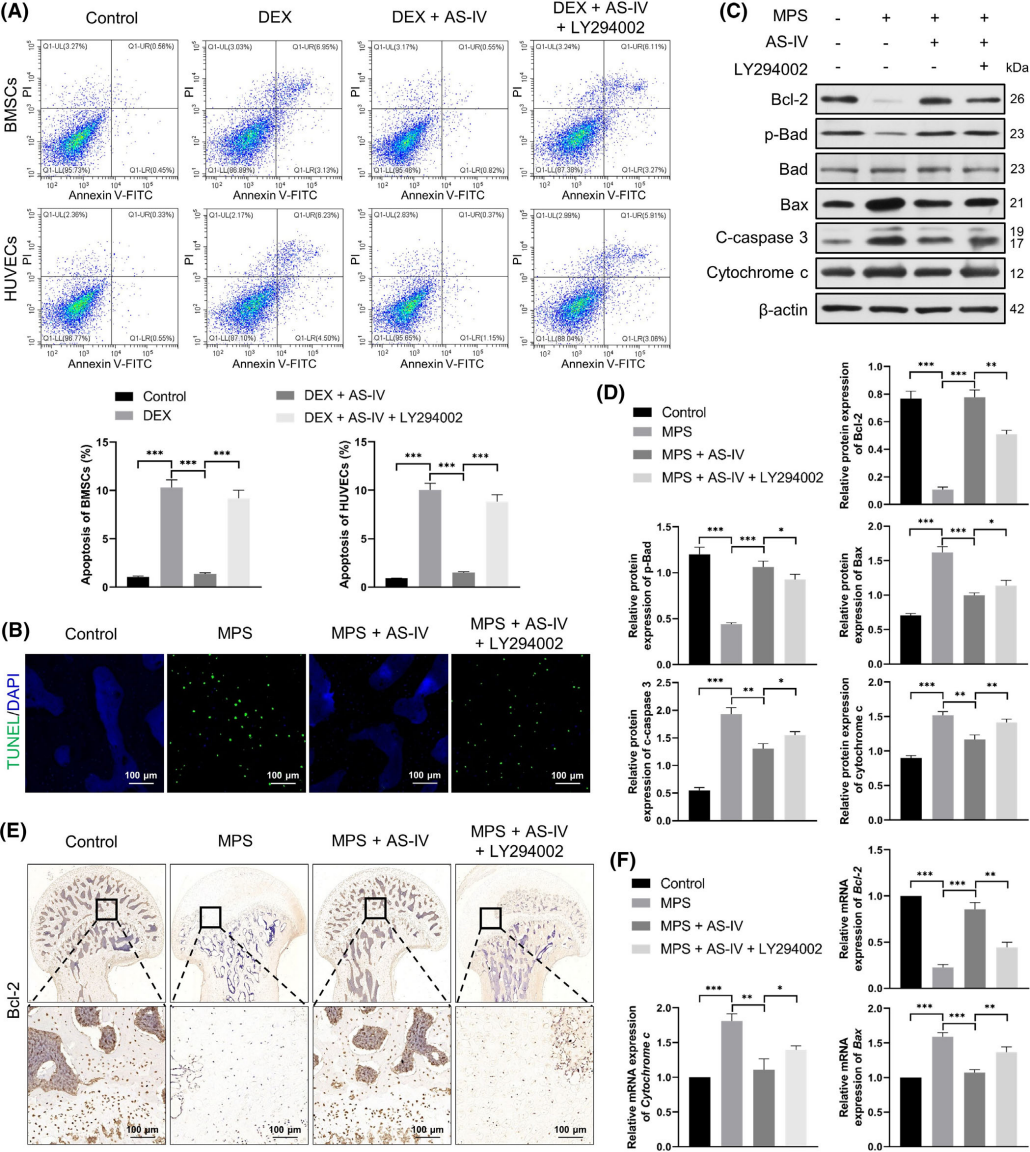
AS‐IV retards GC‐induced apoptosis via the Akt/Bad/Bcl‐2 pathway in vivo and in vitro. (A) Apoptosis of bone marrow mesenchymal stem cells (BMSCs) and human umbilical vein endothelial cells (HUVECs) evaluated by Annexin V‐FITC/propidium iodide double staining and flow cytometry (*n* = 3). (B) Anti‐apoptotic effects of AS‐IV on GC‐induced avascular necrosis of the femoral head (ANFH) evaluated by TdT‐mediated dUTP nick end labelling staining in vivo (scale bar = 100 μm). (C,D) Western blots of p‐Bad, Bad, Bcl‐2, Bax, CC3 and cytochrome‐c protein expression (*n* = 3). (E) Immunohistochemical staining of Bcl‐2 under various conditions (scale bar = 100 μm). (F) Relative mRNA levels of *Bcl‐2*, *Bax* and *Cytochrome c* assessed by qRT‐PCR (*n* = 3). *p < 0.05, **p < 0.01 and ***p < 0.001.

BMSC and HUVEC apoptosis in vitro was determined by Annexin V‐FITC/PI double staining and flow cytometry (Figure 7A); we observed a notable increase of apoptosis in the DEX group compared with that in the control group. Contrarily, AS‐IV inhibited and LY294002 restored cell apoptosis. Furthermore, the number of apoptotic cells was considerably increased in the MPS group compared with that in the control group and decreased by AS‐IV, as observed in the TdT‐mediated dUTP nick end labelling assay (Figure 7B). LY294002 again increased the number of apoptotic cells.

Western blotting (Figure 7C,D), immunohistochemical staining (Figure 7E) and qRT‐PCR (Figure 7F) revealed that the Bcl‐2 and p‐Bad levels were reduced, whereas the Bax, CC3 and cytochrome c levels were increased in the MPS group compared with those in the control group. However, AS‐IV reversed these effects. LY294002 increased the Bcl‐2 and p‐Bad levels, and apparently reduced the Bax, CC3 and cytochrome c levels.

### 3.8 AS‐IV retarded MPS‐induced oxidative stress via the Akt/Nrf2/HO‐1 pathway in vivo

**FIGURE 8:**
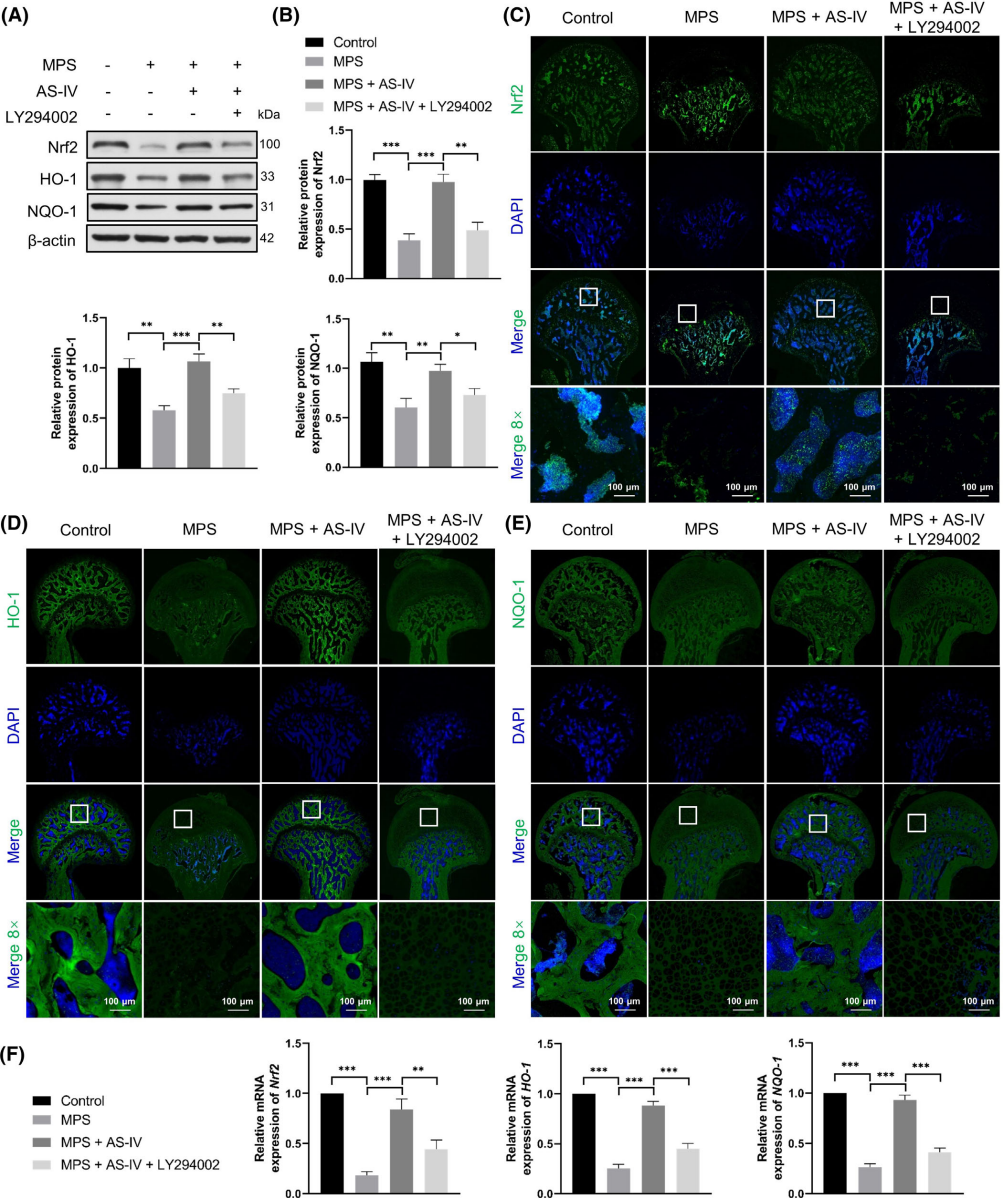
AS‐IV retards MPS‐induced oxidative stress via the Akt/Nrf2/HO‐1 pathway in vivo. (A, B) NQO‐1, Nrf2 and HO‐1 protein levels determined by western blotting (*n* = 3). (C–E) Immunofluorescence staining of NQO‐1, Nrf2 and HO‐1 in each experimental group (scale bar = 100 μm). (F) Relative mRNA levels of *NQO‐1*, *Nrf2* and *HO‐1* assessed by qRT‐PCR (*n* = 3). *p < 0.05, **p < 0.01 and ***p < 0.001.

The role of AS‐IV in GC‐induced oxidative stress was assessed by western blotting (Figure 8A,B), immunofluorescence staining (Figure 8C–E) and qRT‐PCR (Figure 8F). The NQO‐1, Nrf2 and HO‐1 levels were considerably reduced in the MPS group compared with those in the control group, and AS‐IV reversed these effects. Notably, LY294002 reduced the NQO‐1, Nrf2 and HO‐1 levels.

Finally, we studied the effect of AS‐IV on blood coagulation function. Prothrombin time, international normalized ratio and activated partial thromboplastin time were reduced, whereas the plasma fibrinogen level was increased slightly in the MPS group compared with those in the control group. However, AS‐IV and LY294002 showed no obvious effect on these (Figure S5A–D).

No significant difference in body weight was observed among the four rat groups (Figure S6).

**FIGURE 9:**
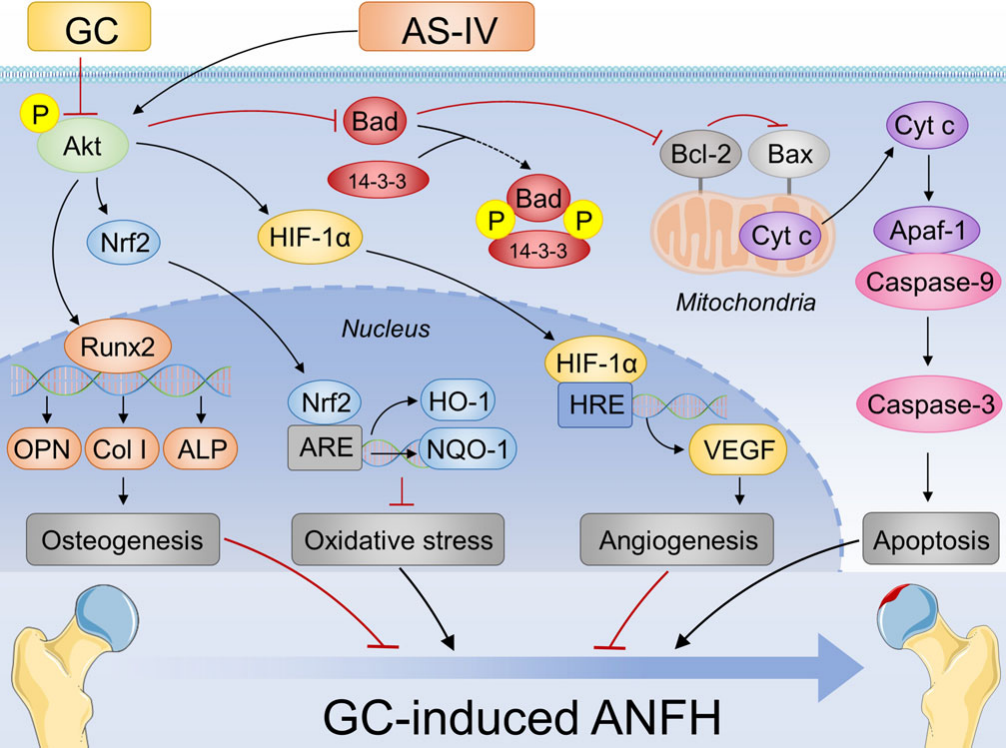
Role of Akt‐mediated signalling pathways in the preventive effects of AS‐IV on GC‐induced ANFH.

Figure 9 shows a schema of the molecular mechanism underlying the preventive effect of AS‐IV on GC‐induced ANFH.

## 4 DISCUSSION

GC‐induced ANFH is a well‐known complication, and it seriously threatens the health of patients. The mechanism of GC‐induced ANFH is complex; it involves osteogenesis inhibition,^7^ vascular injury,^8^ cell apoptosis,^9^ oxidative stress^10^ and mediation via multiple signalling pathways, among which the Akt pathway plays a crucial role.^9^, ^11^, ^12^ AS‐IV, the major active ingredient in *A*. *membranaceus*, has several benefits, including pro‐osteogenic,^22^ pro‐angiogenic,^20^, ^21^ anti‐apoptotic^23^, ^24^ and anti‐oxidative activities.^23^, ^25^

We assessed the role of Akt‐mediated signalling pathways in the ability of AS‐IV to prevent GC‐induced ANFH, which mainly manifests as the promotion of osteogenesis and angiogenesis via the Akt/Runx2 and Akt/HIF‐1α/VEGF pathways, respectively, and as suppression of apoptosis and oxidative stress via the Akt/Bad/Bcl‐2 and Akt/Nrf2/HO‐1 pathways, respectively. The Akt/HIF‐1α/VEGF pathway is a classic vascular signalling pathway. As a highly specific nuclear transcription factor, HIF‐1 plays a crucial role in cell perception and adaptation to changes in oxygen partial pressure in the internal environment.^40^ HIF‐1 comprises α and β subunits; HIF‐1α is the key regulatory and active subunit. Its stability and activity are regulated by the oxygen partial pressure in the internal environment. Under normoxia, HIF‐1α is inhibited by factor‐inhibiting HIF‐1 (FIH‐1) and degraded by the synergistic action of proline hydroxylases and von Hippel–Lindau tumour suppressor (pVHL). However, the activities of FIH‐1 and proline hydroxylases are inhibited in cells exposed to hypoxia. This leads to intracellular HIF‐1α accumulation. HIF‐1α is translocated into the nucleus, where it polymerizes with HIF‐1β, which then binds to the hypoxia response element of the downstream target gene and regulates gene expression.^41^

VEGF is an important target gene of HIF‐1α. The VEGF family mainly comprises VEGFA, VEGFB, VEGFC, VEGFD and placental growth factor. VEGFA promotes new blood vessel formation and increases blood vessel permeability.^42^ We found reduced HIF‐1α, VEGFA, CD31, p‐Akt and vWF levels in the MPS group compared with those in the control group. However, AS‐IV reversed these effects. Moreover, LY294002 apparently reduced their levels, indicating that the repair response in GC‐induced ANFH might be associated with the Akt/HIF‐1α/VEGF pathway, and failure to repair the necrotic femoral head might be related to the lack of VEGF and HIF‐1α. Furthermore, the results implied that AS‐IV rescued GC‐induced inhibition of angiogenesis via the Akt/HIF‐1α/VEGF pathway.

Additionally, apoptosis mediated by an abnormal Akt/Bad/Bcl‐2 pathway is also an important factor for the development of GC‐induced ANFH.^9^ Apoptosis is mainly regulated by Bcl‐2, Bad and Bax. The apoptotic precursor protein Bad, which belongs to the Bcl‐2 family, was discovered as the first protein targeted by Akt, and it is associated with apoptosis. Bad is distributed in the outer mitochondrial membrane, and combines with Bcl‐2 and Bcl‐xL to form a heterodimer that plays an important role in promoting cell apoptosis.^43^ Under normal physiological conditions, activated Akt phosphorylates the Serl36 site on Bad, and p‐Bad stably exists in the cytoplasm by binding to the 14‐3‐3 protein, thereby blocking the dimer formation of Bad and Bcl‐2. Although this prevents Bad from promoting cell apoptosis, it is conducive to the anti‐apoptotic effects of free Bcl‐2. When Akt activation is inhibited, calcineurin can dephosphorylate Bad, which forms a complex with Bcl‐2 and exerts proapoptotic activities.^44^

The anti‐apoptotic protein Bcl‐2, which is mainly found in the outer mitochondrial membrane,^45^ and Bax are the two most representative members of the Bcl‐2 family. A high Bax expression renders cells sensitive to death signals and promotes apoptosis, whereas a high Bcl‐2 expression causes Bcl‐2 to form heterodimers with Bax and inhibits cell apoptosis. Therefore, the ratio of Bcl‐2/Bax plays a crucial role in regulating cellular sensitivity to apoptosis.^46^

Caspase‐3 is downstream of the Bcl‐2 pathway and is the final effector protein of the apoptotic cascade. It forms the core of cell apoptosis, and is known as the death protease. Caspase‐3 usually exists as the inactive caspase‐3 precursor, pro‐caspase‐3. Apoptotic signals activate cellular pro‐caspase‐3 to produce CC3, which has the properties of a mature enzyme and is the main effector enzyme regulating apoptosis.^47^ We found substantially reduced p‐Akt, Bcl‐2 and p‐Bad levels and increased Bax, CC3 and cytochrome c levels in the MPS group compared with those in the control group. However, AS‐IV reversed these effects. Moreover, LY294002 increased the p‐Akt, Bcl‐2 and p‐Bad levels, and decreased the Bax, CC3 and cytochrome c levels, indicating that AS‐IV rescued GC‐induced apoptosis via the Akt/Bad/Bcl‐2 pathway.

The Nrf2/HO‐1 pathway is important in anti‐oxidative processes. The oxidative stress factor Nrf2 is a key redox sensor and the main regulator of the antioxidant response, and exerts cytoprotective effects. Under stress, Nrf2 is phosphorylated and released into the nucleus, where it forms a heterodimer with Maf through the Neh1 domain. It then binds to the antioxidant response element, further promoting the transcription of target genes such as *HO‐1* and *NQO‐1*.^48^ Upon activation, HO‐1, an antioxidant enzyme, can reduce the content of reactive oxygen species in cells and increase the activity of superoxide dismutase and catalase, thereby protecting multiple organs from oxidative stress.^49^ We observed reduced p‐Akt, NQO‐1, Nrf2 and HO‐1 levels in the MPS group compared with those in the control group. AS‐IV reversed these effects. LY294002 apparently reduced the p‐Akt, NQO‐1, Nrf2 and HO‐1 levels. These data indicated that AS‐IV reversed GC‐induced oxidative stress via the Akt/Nrf2/HO‐1 pathway.

Therefore, AS‐IV might be a candidate drug to prevent GC‐induced ANFH, but clinical trials are further required for validation.

In many studies, the optimal concentration of AS‐IV in cell experiments is 20–50 μM.^50^, ^51^, ^52^, ^53^, ^54^, ^55^ Therefore, we selected concentrations of 5, 20, 50 and 100 μM. However, this did not strictly conform to an equal ratio sequence, which was a minor flaw in the design of cell experiments.

Overall, AS‐IV can prevent GC‐induced ANFH by regulating osteogenesis/angiogenesis/apoptosis/oxidative stress via the Akt‐mediated Runx2, HIF‐1α/VEGF, Bad/Bcl‐2 and Nrf2/HO‐1 pathways. However, the crosstalk among the downstream signalling pathways of Akt requires further investigation. Nonetheless, the present findings provide new research targets and strategies to prevent GC‐induced ANFH.

## AUTHOR CONTRIBUTIONS

Xiaowei Yu conceived and designed the study. Haojie Shan, Bojun Cao, Jianzhong Hou, Yiwei Lin, Chenhao Pan, Rongtai Zuo, Fuli Yin, Wenyang Xia, Chaolai Jiang, Tianyi Wu and Zubin Zhou conducted the experiments. Haojie Shan and Yiwei Lin analysed the data and wrote the manuscript. Xiaowei Yu revised the manuscript. All authors read and approved the final version.

## CONFLICT OF INTEREST STATEMENT

The authors have declared that no competing interest exists.

## Supporting information


**Data S1:** Supporting Information

## Data Availability

The datasets used and/or analyzed during the current study are available from the corresponding author upon reasonable request.
