# Supplementary material for: Effects of astragaloside IV on glucocorticoid‐induced avascular necrosis of the femoral head via regulating Akt‐related pathways
Source: Cell Prolif. 2023 Apr 26;56(11):e13485. doi: 10.1111/cpr.13485 (PMC10623974; doi:10.1111/cpr.13485)
Supplement: Supplementary file 1 — Data S1: Supporting Information [file CPR-56-e13485-s001.docx]

**Supplementary Materials**

**Fig. S1.** Structure of AS-IV and Wayne image.

**Fig. S2.** Molecular dynamics simulations of Akt Ser473 microenvironment.

**Fig. S3.**  Down-regulated p-Akt in GC-induced ANFH patients.

**Fig. S4.** Immunofluorescent staining of p-Akt of the femoral head in rats.

**Fig. S5.** AS-IV showed no obvious effect on blood coagulation function.

**Fig. S6.** No significant difference in body weight was observed among the four rat groups.

**Table S1.** Patient characteristics.

**Table S2.** Primer sequences used in qRT-PCR.

**Table S3.** List of enriched AS-IV associated gene sets identified using KEGG pathway analysis.

**Table S4.** Residue-specific protein-ligand interaction energy and its decomposition.


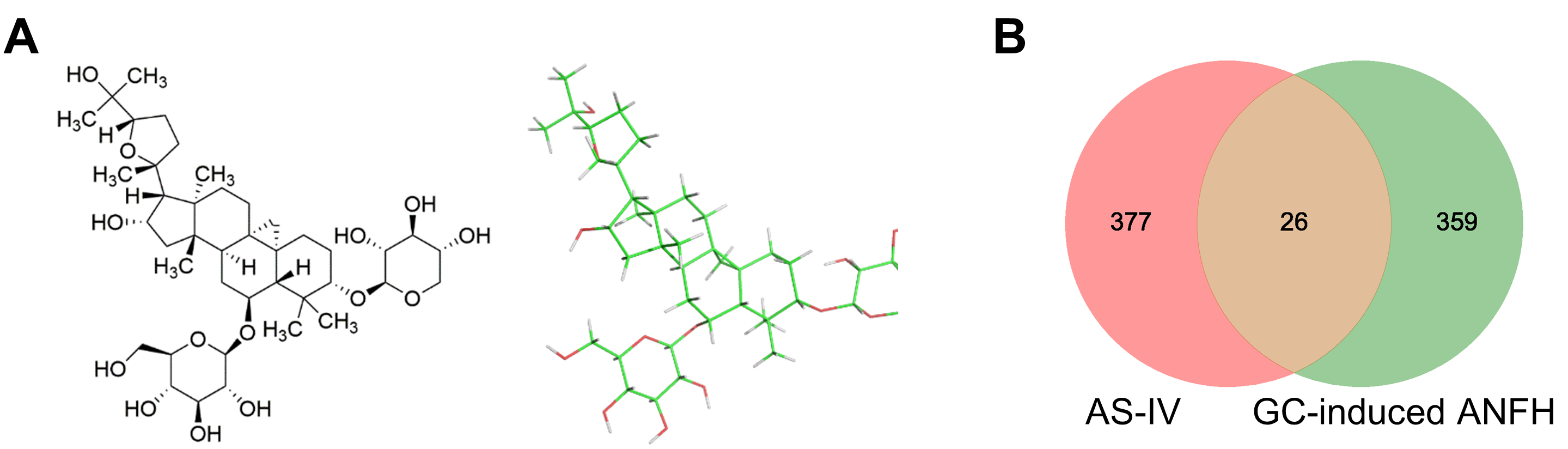


**Fig. S1.**  Structure of AS-IV and Wayne image. (A) Chemical and three-dimensional structure of AS-IV. Molecular formula of C_41_H_68_O_14_ (molecular weight, 784.97 g/mol). (B) Wayne image.


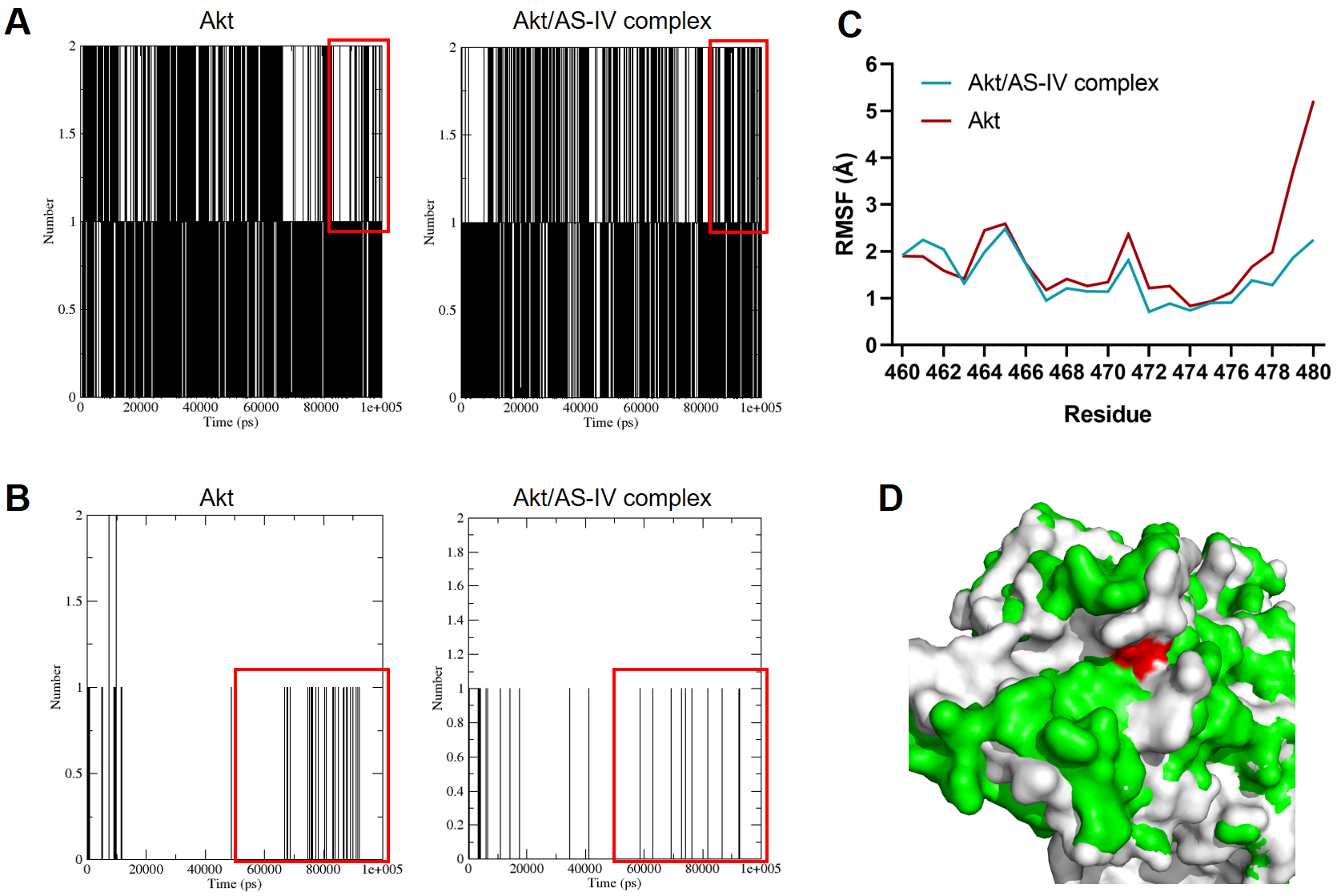


**Fig. S2.** Molecular dynamics simulations of Akt Ser473 microenvironment. (A) Hydrogen bonds between the hydroxyl radical of Akt Ser473 side-chain and the solvent. (B) Hydrogen bonds between the hydroxyl radical of Akt Ser473 side-chain and Akt. (C) RMSF of residues around Ser473. (D) Conformation of the Akt Ser473 microenvironment. The red surface indicates Ser473. The white surface indicates the Akt/AS-IV complex. The green surface indicates Akt.


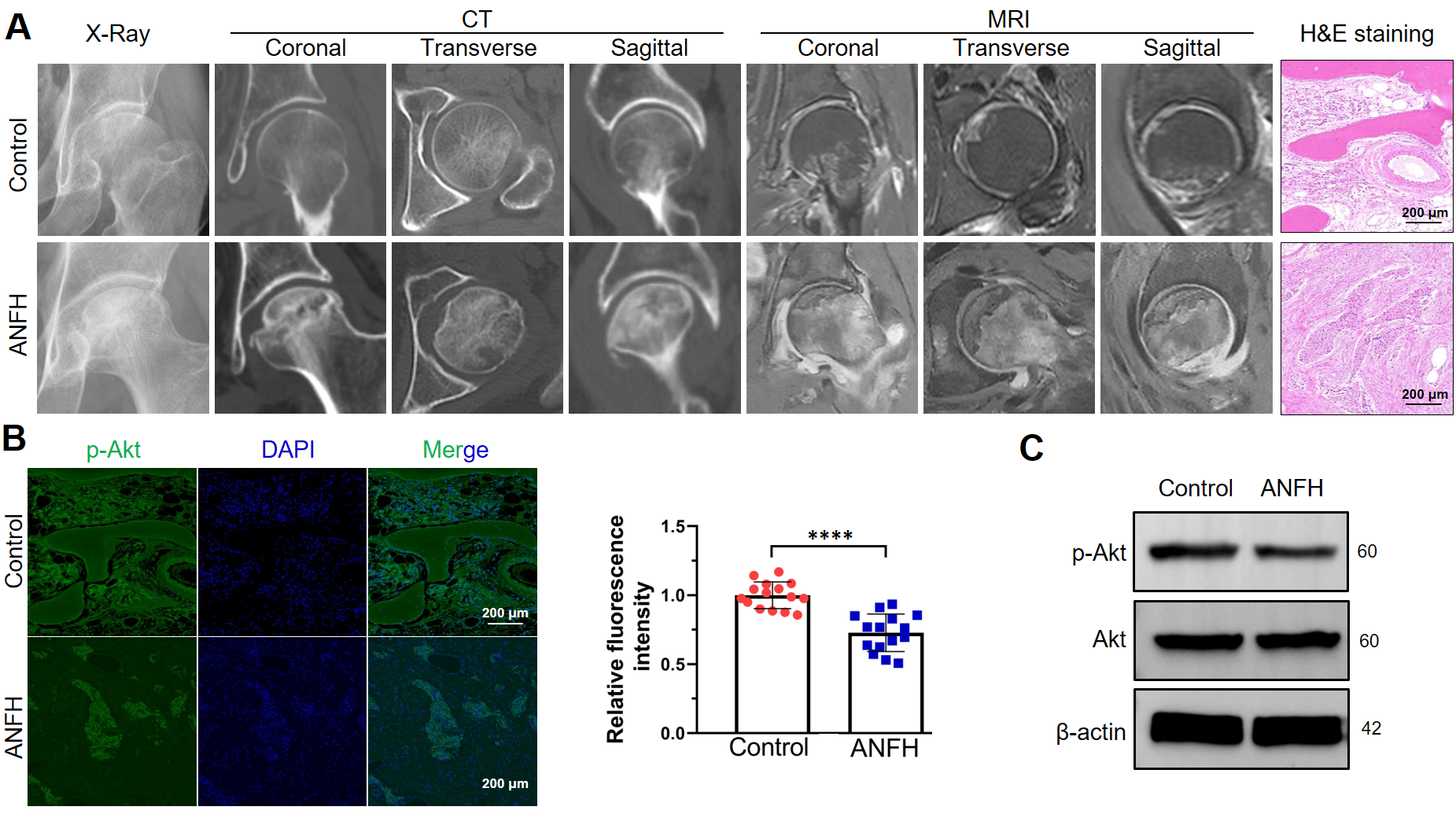


**Fig. S3.**  Downregulation of p-Akt in GC-induced ANFH patients. (A) X-Ray, CT, MRI, and hematoxylin and eosin staining of the femoral head of patients (scale bar = 200 μm). (B) Immunofluorescent staining of p-Akt of the femoral head of patients (scale bar = 200 μm, n = 15). (C) Protein levels of p-Akt and Akt, as assessed by western blotting. *****P* < 0.0001.


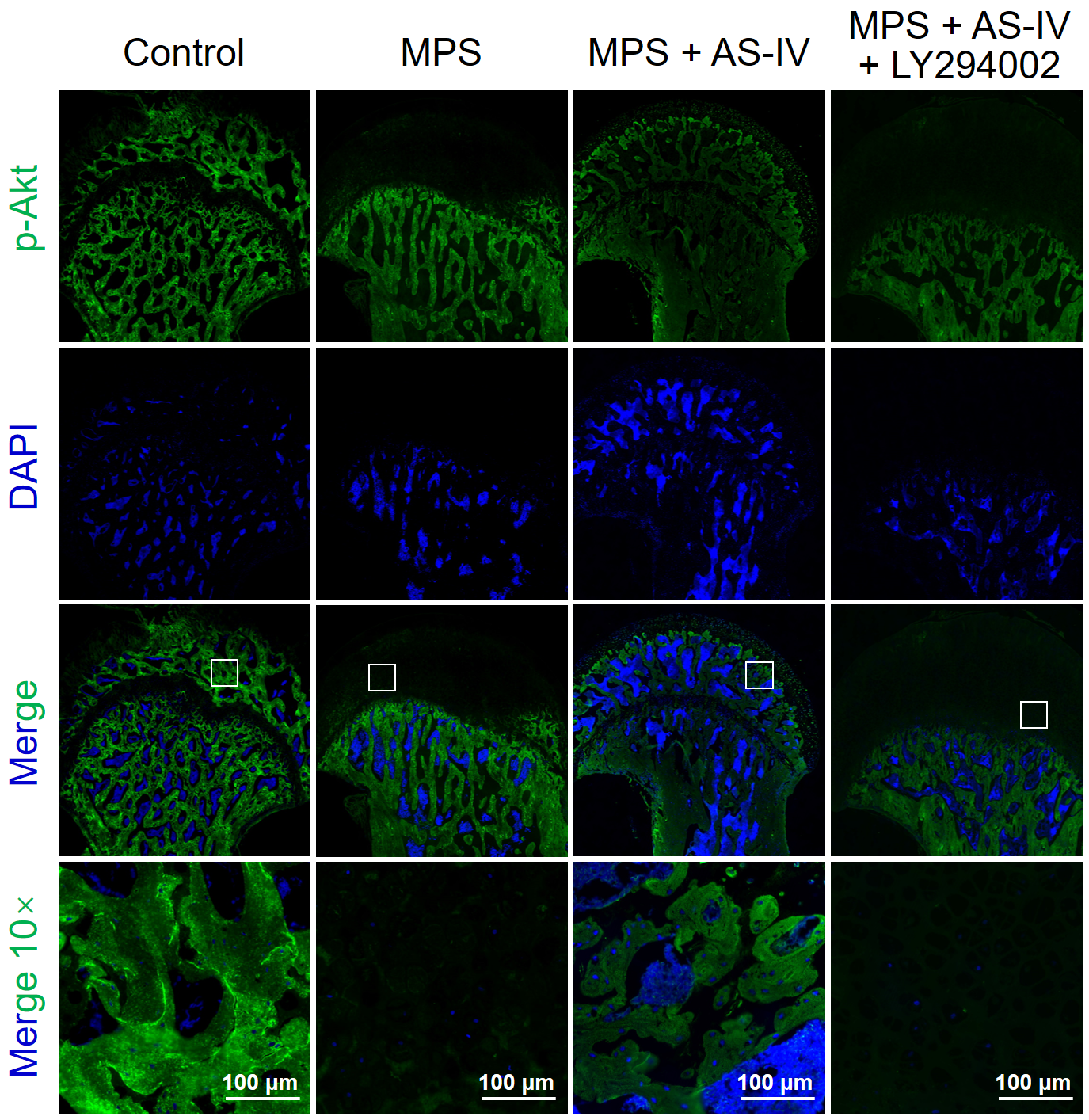


**Fig. S4.** Immunofluorescent staining of p-Akt of the femoral head in rats (scale bar = 100 μm).


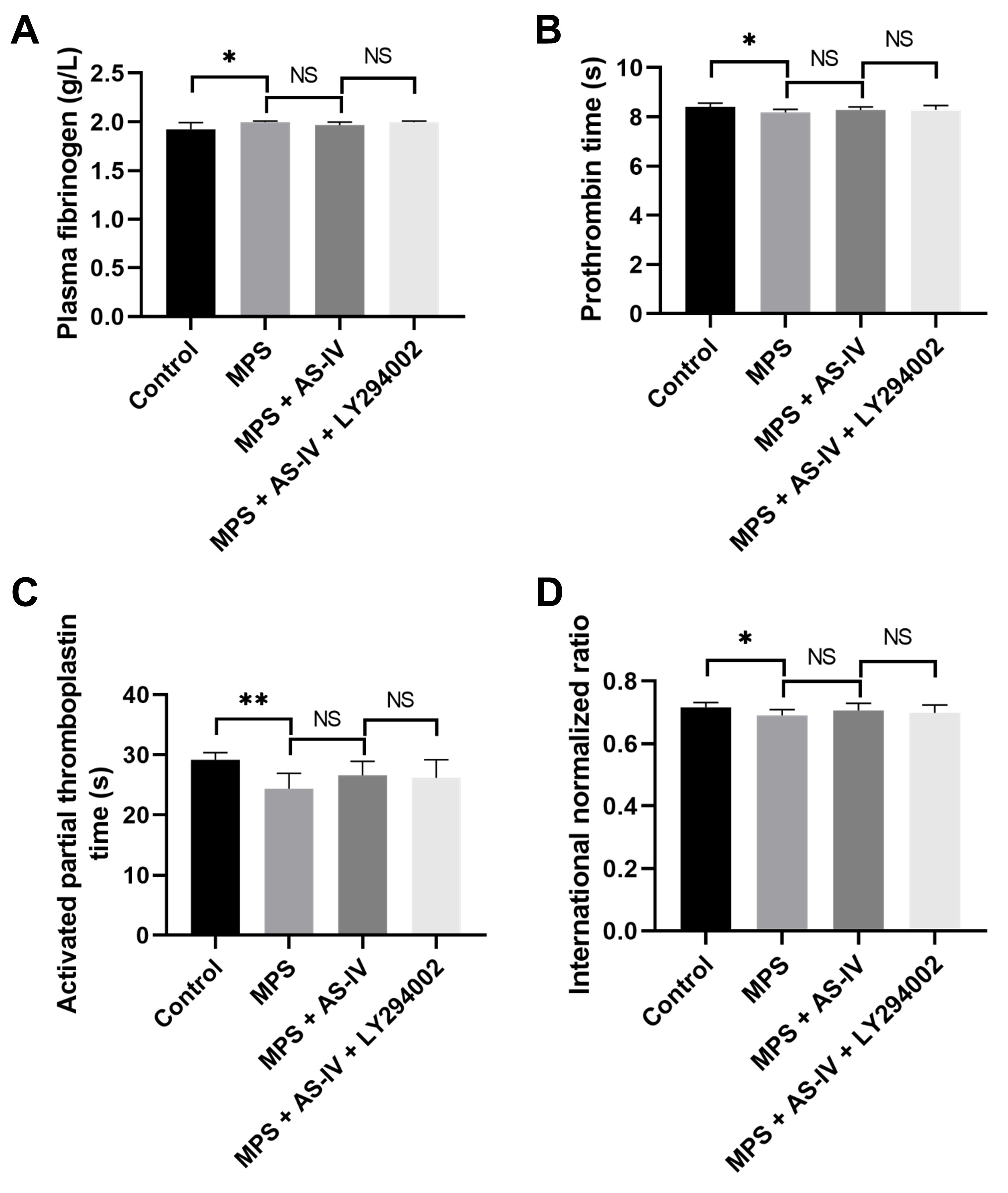


**Fig. S5.** AS-IV showed no obvious effects on blood coagulation function. (A) Plasma fibrinogen level. (B) Prothrombin time. (C) Activated partial thromboplastin time. (D) International normalized ratio. **P* < 0.05, ***P* < 0.01. NS, not significant.

**
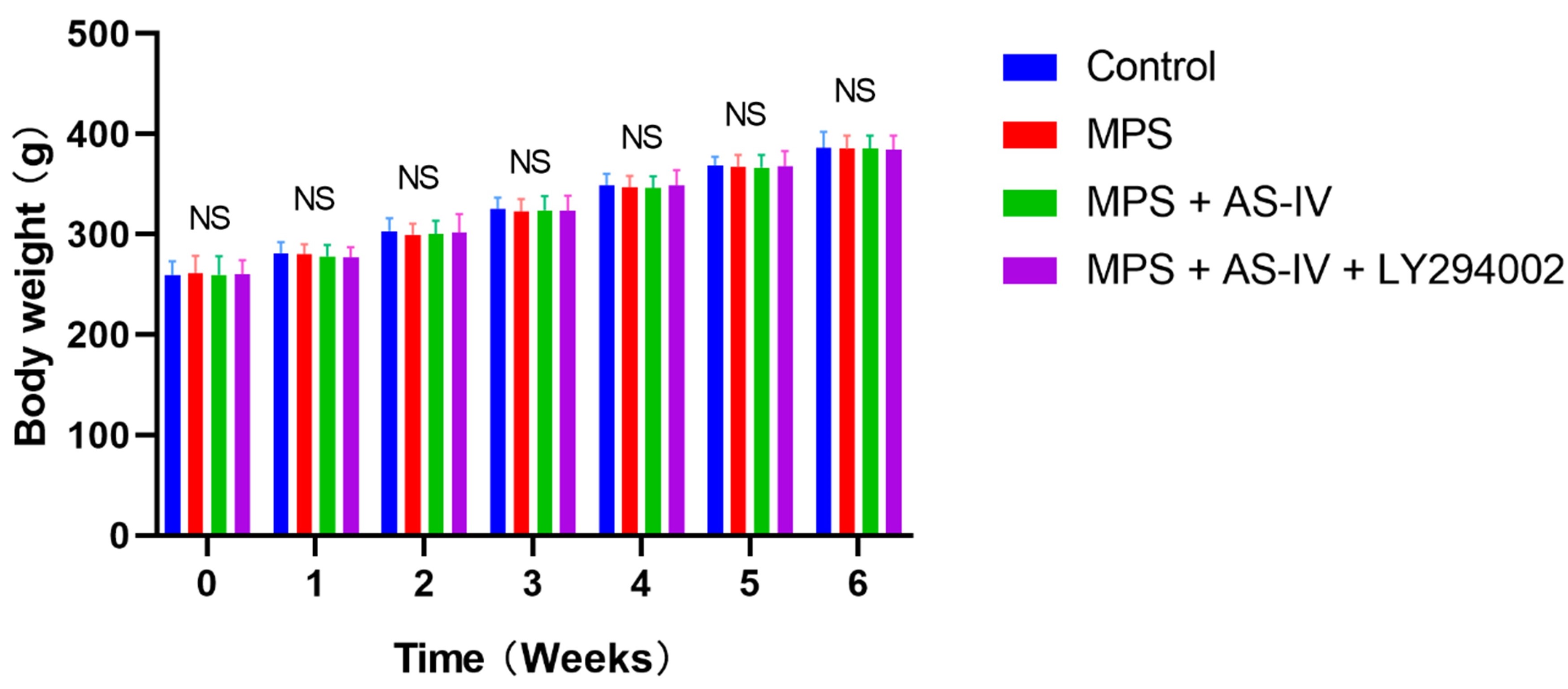
**

**Fig. S6.** No significant difference in body weight was observed among the four rat groups (n = 10). NS, not significant.

**Table S1.** Patient characteristics.

| Control | | |  |  |  |  | GC-induced ANFH | | | |
| --- | --- | --- | --- | --- | --- | --- | --- | --- | --- | --- |
| Sample number | Sex | Age | | | Sample number | | | Sex | Age |  |
| 1 | Male | 70 | | | 1 | | | Male | 65 |  |
| 2 | Male | 65 | | | 2 | | | Male | 83 |  |
| 3 | Male | 70 | | | 3 | | | Male | 60 |  |
| 4 | Male | 63 | | | 4 | | | Male | 70 |  |
| 5 | Male | 70 | | | 5 | | | Male | 65 |  |
| 6 | Male | 75 | | | 6 | | | Male | 76 |  |
| 7 | Male | 69 | | | 7 | | | Male | 74 |  |
| 8 | Female | 64 | | | 8 | | | Male | 71 |  |
| 9 | Female | 65 | | | 9 | | | Female | 68 |  |
| 10 | Female | 70 | | | 10 | | | Female | 62 |  |
| 11 | Female | 72 | | | 11 | | | Female | 78 |  |
| 12 | Female | 70 | | | 12 | | | Female | 79 |  |
| 13 | Female | 67 | | | 13 | | | Female | 69 |  |
| 14 | Female | 65 | | | 14 | | | Female | 82 |  |
| 15 | Female | 81 | | | 15 | | | Female | 56 |  |

**Table S2.** Primer sequences used in qRT-PCR.

| **Primer** | **Forward (5′-3′)** | **Reverse (5′-3′)** |
| --- | --- | --- |
| *HIF-1α* | GCTGCCTCTTCGACAAGCTTA | TTGGTCTTCAGTTTCCGTGTCA |
| *VEGF* | GAGCAGAAAGCCCATGAAGTG | ACTCCAGGGCTTCATCATTGC |
| *ALP* | TGGTACTCGGACAATGAGATGC | GCTCTTCCAAATGCTGATGAGGT |
| *Runx2* | CCTGAACTCAGCACCAAGTCCT | TCAGAGGTGGCAGTGTCATCA |
| *OCN* | AATAGACTCCGCGCTACCTC | GCTAGCTCGTCACAATTGGG |
| *OPN* | GATGAACAGTATCCCGATGCCA | GTCTTCCCGTTGCTGTCCTGA |
| *Collagen I* | GACGCATGGCCAAGAAGACAT | TCTTTGCATAGCACGCCATCG |
| *Bcl-2* | TCTTTGAGTTCGGTGGGGTCA | AGTTCCACAAAGGCATCCCAG |
| *NQO-1* | GCGGTGAGAAGAGCCCTGAT | GCTCCCCTGTGATGTCGTTTC |
| *Nrf2* | TCCCAGCAGGACATGGATTTG | GCTGGCTGAATTGGGAGGAAT |
| *HO-1* | GTGACAGAAGAGGCTAAGACCGC | GAAACTGAGTGTGAGGACCCATC |
| *Bax* | GGCGATGAACTGGACAACAAC | CCCAGTTGAAGTTGCCGTCT |
| *Cytochrome c* | AGGAGGCAAGCATAAGACTGGAC | CCTTTGTTCTTGTTGGCATCTGTG |
| *GAPDH* | CTGGAGAAACCTGCCAAGTATG | GGTGGAAGAATGGGAGTTGCT |

**Table S3.** List of enriched AS-IV associated gene sets identified using KEGG pathway analysis.

| Term | Genes |
| --- | --- |
| hsa05200:Pathways in cancer | TGFB2, HSP90AA1, NOS2, STAT1, MMP1, IGF1, FGF2, ESR1, MMP9, IL2, VEGFA, CCNA2, BMP2, CASP3, **AKT1**, PPARG |
| hsa05205:Proteoglycans in cancer | TGFB2, SRC, CASP3, **AKT1**, IGF1, ESR1, FGF2, MMP9, VEGFA |
| hsa05417:Lipid and atherosclerosis | HSP90AA1, MMP1, SRC, CASP3, CASP1, **AKT1**, PPARG, MMP9 |
| hsa05161:Hepatitis B | CCNA2, TGFB2, STAT1, SRC, CASP3, **AKT1**, MMP9 |
| hsa05152:Tuberculosis | TGFB2, NOS2, STAT1, SRC, VDR, CASP3, **AKT1** |
| hsa05207:Chemical carcinogenesis - receptor activation | HSP90AA1, SRC, VDR, **AKT1**, ESR1, FGF2, VEGFA |
| hsa04926:Relaxin signaling pathway | NOS2, MMP1, SRC, **AKT1**, MMP9, VEGFA |
| hsa01521:EGFR tyrosine kinase inhibitor resistance | SRC, **AKT1**, IGF1, FGF2, VEGFA |
| hsa01522:Endocrine resistance | SRC, **AKT1**, IGF1, ESR1, MMP9 |
| hsa04933:AGE-RAGE signaling pathway in diabetic complications | TGFB2, STAT1, CASP3, **AKT1**, VEGFA |
| hsa05167:Kaposi sarcoma-associated herpesvirus infection | STAT1, SRC, CASP3, **AKT1**, FGF2, VEGFA |
| hsa04625:C-type lectin receptor signaling pathway | STAT1, SRC, CASP1, **AKT1**, IL2 |
| hsa05219:Bladder cancer | MMP1, SRC, MMP9, VEGFA |
| hsa05145:Toxoplasmosis | TGFB2, NOS2, STAT1, CASP3, **AKT1** |
| hsa04919:Thyroid hormone signaling pathway | KAT2B, STAT1, SRC, **AKT1**, ESR1 |
| hsa05166:Human T-cell leukemia virus 1 infection | CCNA2, KAT2B, TGFB2, MMP7, **AKT1**, IL2 |
| hsa04915:Estrogen signaling pathway | HSP90AA1, SRC, **AKT1**, ESR1, MMP9 |
| hsa05418:Fluid shear stress and atherosclerosis | HSP90AA1, SRC, **AKT1**, MMP9, VEGFA |
| hsa04917:Prolactin signaling pathway | STAT1, SRC, **AKT1**, ESR1 |
| hsa03320:PPAR signaling pathway | MMP1, APOA2, PPARG, ACADM |
| hsa05212:Pancreatic cancer | TGFB2, STAT1, **AKT1**, VEGFA |
| hsa04010:MAPK signaling pathway | TGFB2, CASP3, **AKT1**, IGF1, FGF2, VEGFA |
| hsa04657:IL-17 signaling pathway | HSP90AA1, MMP1, CASP3, MMP9 |
| hsa05203:Viral carcinogenesis | CCNA2, KAT2B, SRC, CASP3, HDAC8 |
| hsa05215:Prostate cancer | HSP90AA1, **AKT1**, IGF1, MMP9 |
| hsa04015:Rap1 signaling pathway | SRC, **AKT1**, IGF1, FGF2, VEGFA |
| hsa05142:Chagas disease | TGFB2, NOS2, **AKT1**, IL2 |
| hsa04914:Progesterone-mediated oocyte maturation | CCNA2, HSP90AA1, **AKT1**, IGF1 |
| hsa04151:PI3K-Akt signaling pathway | HSP90AA1, **AKT1**, IGF1, FGF2, IL2, VEGFA |
| hsa04066:HIF-1 signaling pathway | NOS2, **AKT1**, IGF1, VEGFA |
| hsa04152:AMPK signaling pathway | CCNA2, **AKT1**, PPARG, IGF1 |
| hsa04380:Osteoclast differentiation | TGFB2, STAT1, **AKT1**, PPARG |
| hsa05135:Yersinia infection | SRC, CASP1, **AKT1**, IL2 |
| hsa05162:Measles | STAT1, CASP3, **AKT1**, IL2 |
| hsa05224:Breast cancer | **AKT1**, IGF1, ESR1, FGF2 |
| hsa04370:VEGF signaling pathway | SRC, **AKT1**, VEGFA |
| hsa05164:Influenza A | STAT1, CASP3, CASP1, **AKT1** |
| hsa05165:Human papillomavirus infection | CCNA2, STAT1, CASP3, **AKT1**, VEGFA |
| hsa05321:Inflammatory bowel disease | TGFB2, STAT1, IL2 |
| hsa05211:Renal cell carcinoma | TGFB2, **AKT1**, VEGFA |
| hsa04613:Neutrophil extracellular trap formation | SRC, CASP1, **AKT1**, HDAC8 |
| hsa05202:Transcriptional misregulation in cancer | CCNA2, PPARG, IGF1, MMP9 |
| hsa05218:Melanoma | **AKT1**, IGF1, FGF2 |
| hsa04510:Focal adhesion | SRC, **AKT1**, IGF1, VEGFA |
| hsa05169:Epstein-Barr virus infection | CCNA2, STAT1, CASP3, **AKT1** |
| hsa05133:Pertussis | NOS2, CASP3, CASP1 |
| hsa05140:Leishmaniasis | TGFB2, NOS2, STAT1 |
| hsa05210:Colorectal cancer | TGFB2, CASP3, **AKT1** |
| hsa05163:Human cytomegalovirus infection | SRC, CASP3, **AKT1**, VEGFA |
| hsa04211:Longevity regulating pathway | **AKT1**, PPARG, IGF1 |
| hsa05222:Small cell lung cancer | NOS2, CASP3, **AKT1** |
| hsa05171:Coronavirus disease - COVID-19 | STAT1, MMP1, CASP1, IL2 |
| hsa05323:Rheumatoid arthritis | TGFB2, MMP1, VEGFA |
| hsa04014:Ras signaling pathway | **AKT1**, IGF1, FGF2, VEGFA |
| hsa05132:Salmonella infection | HSP90AA1, CASP3, CASP1, **AKT1** |
| hsa05146:Amoebiasis | TGFB2, NOS2, CASP3 |
| hsa04659:Th17 cell differentiation | HSP90AA1, STAT1, IL2 |
| hsa04668:TNF signaling pathway | CASP3, **AKT1**, MMP9 |
| hsa04935:Growth hormone synthesis, secretion and action | STAT1, **AKT1**, IGF1 |
| hsa04068:FoxO signaling pathway | TGFB2, **AKT1**, IGF1 |
| hsa05206:MicroRNAs in cancer | TGFB2, CASP3, MMP9, VEGFA |
| hsa04936:Alcoholic liver disease | CASP3, **AKT1**, ACADM |
| hsa04550:Signaling pathways regulating pluripotency of stem cells | **AKT1**, IGF1, FGF2 |
| hsa05226:Gastric cancer | TGFB2, **AKT1**, FGF2 |
| hsa04932:Non-alcoholic fatty liver disease | CASP3, **AKT1**, PPARG |
| hsa04218:Cellular senescence | CCNA2, TGFB2, **AKT1** |
| hsa05160:Hepatitis C | STAT1, CASP3, **AKT1** |
| hsa04217:Necroptosis | HSP90AA1, STAT1, CASP1 |
| hsa04630:JAK-STAT signaling pathway | STAT1, **AKT1**, IL2 |

**Table S4.** Residue-specific protein-ligand interaction energy and its decomposition.

| Location | van der Waals  (Δ*E_vdw_*) | Electrostatic  (Δ*E_ele_*) | Polar Solv. (Δ*E_pol_*) | Nonpolar Solv. (Δ*E_npol_*) | Total  (Δ*G_total_*) |
| --- | --- | --- | --- | --- | --- |
| R VAL147 | -0.000153846 | 0.080307692 | -0.079538462 | 0 | 0.000615385 |
| R THR148 | -0.000115385 | -0.000769231 | 0.001 | 0 | 0.000115385 |
| R MET149 | -0.0005 | -0.000346154 | 0.000769231 | 0 | -0.000076923 |
| R ASN150 | -0.000269231 | 0.000076923 | -0.000230769 | 0 | -0.000423077 |
| R ASP151 | -0.000115385 | -0.072730769 | 0.072615385 | 0 | -0.000230769 |
| R PHE152 | -0.000461538 | 0.002807692 | -0.002884615 | 0 | -0.000538462 |
| R ASP153 | -0.000230769 | -0.065884615 | 0.064923077 | 0 | -0.001192308 |
| R TYR154 | -0.000461538 | 0.002730769 | -0.001 | 0 | 0.001269231 |
| R LEU155 | -0.000653846 | 0.003692308 | 0.000192308 | 0 | 0.003230769 |
| R LYS156 | -0.0015 | 0.030846154 | -0.019923077 | 0 | 0.009423077 |
| R LEU157 | -0.006884615 | -0.008384615 | 0.0165 | 0 | 0.001230769 |
| R LEU158 | -0.009346154 | 0.013307692 | -0.034230769 | 0 | -0.030269231 |
| R GLY159 | -0.016615385 | -0.072576923 | 0.026384615 | 0 | -0.062807692 |
| R LYS160 | -0.100730769 | -0.139807692 | 0.323423077 | -0.004229723 | 0.078654892 |
| R GLY161 | -0.138423077 | -0.102192308 | 0.090307692 | -0.014781323 | -0.165089015 |
| R THR162 | -1.177076923 | -0.526307692 | 1.384730769 | -0.344101015 | -0.662754862 |
| R PHE163 | -0.790115385 | -0.267807692 | 0.520346154 | -0.138508062 | -0.676084985 |
| R GLY164 | -0.029730769 | 0.042192308 | 0.053153846 | 0 | 0.065615385 |
| R LYS165 | -0.012192308 | -0.042692308 | 0.068192308 | 0 | 0.013307692 |
| R VAL166 | -0.013653846 | 0.018192308 | -0.041884615 | 0 | -0.037346154 |
| R ILE167 | -0.002692308 | -0.007884615 | 0.010576923 | 0 | -3.979722534 |
| R LEU168 | -0.001346154 | 0.007 | -0.005 | 0 | 0.000653846 |
| R VAL169 | -0.000730769 | -0.003730769 | 0.004 | 0 | -0.000461538 |
| R ARG170 | -0.000230769 | 0.076692308 | -0.076423077 | 0 | 0.000038462 |
| R GLU171 | -0.000192308 | -0.091423077 | 0.090653846 | 0 | -0.000961538 |
| R LYS172 | -0.000153846 | 0.0605 | -0.060230769 | 0 | 0.000115385 |
| R ALA173 | 0 | 0.001653846 | -0.001423077 | 0 | 0.000230769 |
| R THR174 | 0 | -0.001576923 | 0.001461538 | 0 | -0.000115385 |
| R GLY175 | -0.000115385 | -0.000961538 | 0.001192308 | 0 | 0.000115385 |
| R ARG176 | -0.000384615 | 0.132153846 | -0.130884615 | 0 | 0.000884615 |
| R TYR177 | -0.000384615 | -0.004192308 | 0.006461538 | 0 | 0.001884615 |
| R TYR178 | -0.000923077 | 0.008769231 | -0.006115385 | 0 | 0.001730769 |
| R ALA179 | -0.001576923 | -0.003807692 | 0.006884615 | 0 | 0.0015 |
| R MET180 | -0.002269231 | 0.013923077 | -0.011115385 | 0 | 0.000538462 |
| R LYS181 | -0.0245 | 0.535423077 | -0.664884615 | 0 | -0.153961538 |
| R ILE182 | -0.006076923 | 0.004653846 | 0.002230769 | 0 | 0.000807692 |
| R LEU183 | -0.024615385 | 0.021423077 | -0.020538462 | 0 | -0.023730769 |
| R ARG184 | -0.010846154 | 0.017923077 | 0.031230769 | 0 | 0.038307692 |
| R LYS185 | -0.004269231 | 0.075961538 | -0.051653846 | 0 | 0.020038462 |
| R GLU186 | -0.002769231 | -0.030923077 | 0.050384615 | 0 | 0.016692308 |
| R VAL187 | -0.008961538 | -0.007769231 | 0.024884615 | 0 | 0.008153846 |
| R ILE188 | -0.004653846 | 0.001692308 | 0.010538462 | 0 | 0.007576923 |
| R ILE189 | -0.004923077 | 0.011153846 | 0.002846154 | 0 | 0.009076923 |
| R ALA190 | -0.019461538 | -0.038846154 | 0.063346154 | 0 | 0.005038462 |
| R LYS191 | -0.050307692 | -0.136884615 | 0.171269231 | 0 | -0.015923077 |
| R ASP192 | -0.466923077 | -0.682769231 | 0.899076923 | -0.113631785 | -0.364247169 |
| R GLU193 | -0.322115385 | -0.339653846 | 1.083153846 | -0.034249846 | 0.387134769 |
| R VAL194 | -0.053115385 | -0.053961538 | 0.024230769 | 0 | -0.082846154 |
| R ALA195 | -0.285576923 | -0.049 | -0.186615385 | -0.038954492 | -0.5601468 |
| R HIE196 | -1.296346154 | -1.293115385 | 1.787307692 | -0.223476092 | -1.025629938 |
| R THR197 | -0.062923077 | -0.003115385 | 0.108538462 | 0 | 0.0425 |
| R VAL198 | -0.031423077 | -0.024692308 | 0.030192308 | 0 | -0.025923077 |
| R THR199 | -0.2345 | -0.089423077 | 0.236653846 | -0.038873077 | -0.126142308 |
| R GLU200 | -0.270076923 | -1.277538462 | 2.4815 | -0.020428615 | 0.913456 |
| R SER201 | -0.015115385 | 0.008807692 | 0.061269231 | 0 | 0.054961538 |
| R ARG202 | -0.020153846 | 0.148230769 | -0.050346154 | 0 | 0.077730769 |
| R VAL203 | -0.033346154 | 0.052884615 | -0.032346154 | 0 | -0.012807692 |
| R LEU204 | -0.013153846 | 0.022884615 | -0.012346154 | 0 | -0.002615385 |
| R GLN205 | -0.005038462 | 0.036192308 | 0.002 | 0 | 0.033153846 |
| R ASN206 | -0.002192308 | 0.015269231 | 0.001423077 | 0 | 0.0145 |
| R THR207 | -0.001730769 | -0.008692308 | 0.016461538 | 0 | 0.006038462 |
| R ARG208 | -0.000692308 | 0.191384615 | -0.1865 | 0 | 0.004192308 |
| R HIE209 | -0.001307692 | -0.004076923 | 0.008038462 | 0 | 0.002653846 |
| R PRO210 | -0.000730769 | -0.007307692 | 0.009730769 | 0 | 0.001692308 |
| R PHE211 | -0.001961538 | -0.016692308 | 0.014076923 | 0 | -0.004576923 |
| R LEU212 | -0.004230769 | 0.028576923 | -0.014346154 | 0 | 0.01 |
| R THR213 | -0.004 | 0.004346154 | 0.008769231 | 0 | 0.009115385 |
| R ALA214 | -0.001 | -0.000692308 | 0.002961538 | 0 | 0.001269231 |
| R LEU215 | -0.002 | 0.004153846 | 0.001576923 | 0 | 0.003730769 |
| R LYS216 | -0.000653846 | 0.163730769 | -0.158692308 | 0 | 0.004384615 |
| R TYR217 | -0.000653846 | -0.009884615 | 0.01 | 0 | -0.000538462 |
| R ALA218 | -0.000730769 | 0.0045 | -0.002961538 | 0 | 0.000807692 |
| R PHE219 | -0.001 | -0.008692308 | 0.009615385 | 0 | -0.000076923 |
| R GLN220 | -0.000884615 | 0.002807692 | 0.002307692 | 0 | 0.004230769 |
| R THR221 | -0.000615385 | -0.005884615 | 0.005923077 | 0 | -0.000576923 |
| R HIE222 | -0.000769231 | -0.0025 | 0.004807692 | 0 | 0.001538462 |
| R ASP223 | -0.001153846 | -0.052038462 | 0.056653846 | 0 | 0.003461538 |
| R ARG224 | -0.002307692 | 0.06 | -0.044461538 | 0 | 0.013230769 |
| R LEU225 | -0.004769231 | 0.001615385 | 0.001153846 | 0 | -0.002 |
| R CYS226 | -0.0015 | 0.001230769 | 0.006307692 | 0 | 0.006038462 |
| R PHE227 | -0.005807692 | 0.002076923 | -0.003730769 | 0 | -0.007461538 |
| R VAL228 | -0.001346154 | -0.003961538 | 0.006615385 | 0 | 0.001307692 |
| R MET229 | -0.004 | 0.027307692 | -0.020346154 | 0 | 0.002961538 |
| R GLU230 | -0.001192308 | -0.238307692 | 0.241692308 | 0 | 0.002192308 |
| R TYR231 | -0.001807692 | 0.003423077 | 0.002384615 | 0 | 0.004 |
| R ALA232 | -0.000923077 | 0.003653846 | 0.002461538 | 0 | 0.005192308 |
| R ASN233 | -0.000692308 | -0.002 | 0.006384615 | 0 | 0.003692308 |
| R GLY234 | -0.000538462 | 0.002769231 | 0.002038462 | 0 | 0.004269231 |
| R GLY235 | -0.001076923 | -0.017192308 | 0.021 | 0 | 0.002730769 |
| R GLU236 | -0.008038462 | -0.151346154 | 0.170692308 | 0 | 0.011307692 |
| R LEU237 | -0.006192308 | 0.036269231 | -0.027961538 | 0 | 0.002115385 |
| R PHE238 | -0.034115385 | 0.031807692 | 0.023307692 | 0 | 0.021 |
| R PHE239 | -0.003115385 | 0.003384615 | 0.007346154 | 0 | 0.007615385 |
| R HIE240 | -0.001730769 | 0.021384615 | -0.012307692 | 0 | 0.007346154 |
| R LEU241 | -0.003346154 | 0.024538462 | -0.016192308 | 0 | 0.005 |
| R SER242 | -0.001730769 | 0.018961538 | -0.009884615 | 0 | 0.007346154 |
| R ARG243 | -0.001115385 | 0.164807692 | -0.157692308 | 0 | 0.006 |
| R GLU244 | -0.000576923 | -0.175538462 | 0.177423077 | 0 | 0.001307692 |
| R ARG245 | -0.001346154 | 0.180038462 | -0.169192308 | 0 | 0.0095 |
| R VAL246 | -0.000538462 | 0.008884615 | -0.004846154 | 0 | 0.0035 |
| R PHE247 | -0.001269231 | 0.001807692 | -0.006153846 | 0 | -0.005615385 |
| R THR248 | -0.000230769 | -0.016038462 | 0.015115385 | 0 | -0.001153846 |
| R GLU249 | -0.000384615 | -0.190961538 | 0.189807692 | 0 | -0.001538462 |
| R GLU250 | -0.000192308 | -0.189692308 | 0.188807692 | 0 | -0.001076923 |
| R ARG251 | -0.000730769 | 0.227269231 | -0.2185 | 0 | 0.008038462 |
| R ALA252 | -0.000576923 | -0.013923077 | 0.015576923 | 0 | 0.001076923 |
| R ARG253 | -0.000615385 | 0.187692308 | -0.186576923 | 0 | 0.0005 |
| R PHE254 | -0.0005 | -0.008384615 | 0.009923077 | 0 | 0.001038462 |
| R TYR255 | -0.001884615 | -0.016230769 | 0.014153846 | 0 | -0.003961538 |
| R GLY256 | -0.000653846 | -0.018807692 | 0.018730769 | 0 | -0.000730769 |
| R ALA257 | -0.000538462 | -0.008846154 | 0.009923077 | 0 | 0.000538462 |
| R GLU258 | -0.001269231 | -0.314346154 | 0.314538462 | 0 | -0.001076923 |
| R ILE259 | -0.005961538 | -0.005807692 | 0.006730769 | 0 | -0.005038462 |
| R VAL260 | -0.002 | -0.013615385 | 0.017 | 0 | 0.001384615 |
| R SER261 | -0.001 | -0.001692308 | 0.006115385 | 0 | 0.003423077 |
| R ALA262 | -0.002269231 | 0.007730769 | 0.003 | 0 | 0.008461538 |
| R LEU263 | -0.011730769 | 0.010115385 | -0.013923077 | 0 | -0.015538462 |
| R GLU264 | -0.001807692 | -0.273230769 | 0.271961538 | 0 | -0.003076923 |
| R TYR265 | -0.002538462 | 0.021423077 | -0.012346154 | 0 | 0.006538462 |
| R LEU266 | -0.008884615 | 0.044923077 | -0.039461538 | 0 | -0.003423077 |
| R HIE267 | -0.010884615 | 0.101961538 | -0.078115385 | 0 | 0.012961538 |
| R SER268 | -0.001038462 | 0.021192308 | -0.013384615 | 0 | 0.006769231 |
| R ARG269 | -0.002884615 | 0.253307692 | -0.2325 | 0 | 0.017923077 |
| R ASP270 | -0.004576923 | -0.128461538 | 0.164346154 | 0 | 0.031307692 |
| R VAL271 | -0.015076923 | -0.015615385 | 0.036153846 | 0 | 0.005461538 |
| R VAL272 | -0.082615385 | 0.020192308 | -0.037 | 0 | -0.099423077 |
| R TYR273 | -0.174538462 | -0.162538462 | 0.181692308 | 0 | -0.155384615 |
| R ARG274 | -3.291807692 | 0.625076923 | 1.334692308 | -0.499332185 | -1.831370646 |
| R ASP275 | -0.942538462 | -6.718576923 | 9.287346154 | -0.166688862 | 1.459541908 |
| R ILE276 | -0.309730769 | 0.001730769 | -0.011769231 | -0.000265569 | -0.3200348 |
| R LYS277 | -1.832346154 | -0.016115385 | 0.399115385 | -0.328681385 | -1.778027538 |
| R LEU278 | -0.081538462 | 0.128961538 | -0.318153846 | 0 | -0.270730769 |
| R GLU279 | 0.541307692 | -14.12365385 | 11.99730769 | -0.101025969 | -1.686064431 |
| R ASN280 | -0.580846154 | -0.120115385 | 0.698269231 | -0.051063231 | -0.053755538 |
| R LEU281 | -0.025884615 | 0.012961538 | -0.010307692 | 0 | -0.023230769 |
| R MET282 | -0.007076923 | -0.018307692 | 0.029730769 | 0 | 0.004346154 |
| R LEU283 | -0.001769231 | -0.001807692 | 0.004230769 | 0 | 0.000653846 |
| R ASP284 | -0.000730769 | -0.228730769 | 0.226538462 | 0 | -0.002923077 |
| R LYS285 | -0.000307692 | 0.160615385 | -0.159884615 | 0 | 0.000423077 |
| R ASP286 | -0.000153846 | -0.166615385 | 0.166115385 | 0 | -0.000653846 |
| R GLY287 | -0.000307692 | -0.003692308 | 0.007115385 | 0 | 0.003115385 |
| R HIE288 | -0.000692308 | -0.001 | 0.002730769 | 0 | 0.001038462 |
| R ILE289 | -0.002653846 | 0.018615385 | -0.002615385 | 0 | 0.013346154 |
| R LYS290 | -0.003653846 | 0.305307692 | -0.275230769 | 0 | 0.026423077 |
| R ILE291 | -0.014615385 | 0.056153846 | -0.068115385 | 0 | -0.026576923 |
| R THR292 | -0.035230769 | -0.094769231 | -0.006269231 | 0 | -0.136269231 |
| R ASP293 | -0.107076923 | -0.731961538 | 0.852423077 | 0 | 0.013384615 |
| R PHE294 | -0.159230769 | -0.186038462 | -0.089538462 | -0.000531138 | -0.435338831 |
| R GLY295 | -0.244384615 | 0.002038462 | -0.175615385 | -0.006675231 | -0.424636769 |
| R LEU296 | -0.075576923 | 0.041076923 | -0.045115385 | 0 | -0.079615385 |
| R CYS297 | -0.697 | -0.517115385 | 0.726615385 | -0.093650123 | -0.581150123 |
| R LYS298 | -0.031653846 | -0.038923077 | 0.188692308 | 0 | 0.118115385 |
| R GLU299 | -0.038961538 | 0.109230769 | -0.054576923 | 0 | 0.015692308 |
| R GLY300 | -0.173653846 | -0.081115385 | 0.268115385 | -0.020156954 | -0.0068108 |
| R ILE301 | -0.573538462 | 0.026384615 | 0.071961538 | -0.122795169 | -0.597987477 |
| R SER302 | -0.020769231 | -0.008307692 | 0.044461538 | 0 | 0.015384615 |
| R ASP303 | -0.006653846 | 0.081807692 | -0.033807692 | 0 | 0.041346154 |
| R GLY304 | -0.002423077 | -0.0035 | 0.015692308 | 0 | 0.009769231 |
| R ALA305 | -0.009076923 | -0.002038462 | 0.035153846 | 0 | 0.024038462 |
| R THR306 | -0.024576923 | -0.0235 | 0.075923077 | 0 | 0.027846154 |
| R MET307 | -0.038846154 | 0.025269231 | 0.049346154 | 0 | 0.035769231 |
| R LYS308 | -0.840884615 | 0.366769231 | 0.323653846 | -0.117686215 | -0.268147754 |
| R THR309 | -0.061269231 | -0.018615385 | 0.067961538 | 0 | -0.011923077 |
| R PHE310 | -0.039461538 | 0.003769231 | 0.033076923 | 0 | -0.002615385 |
| R CYS311 | -0.656115385 | 0.126653846 | 0.133961538 | -0.054516738 | -0.450016738 |
| R GLY312 | -0.549461538 | 0.765769231 | -1.315730769 | -0.025572462 | -1.124995538 |
| R THR313 | -1.088269231 | 1.387576923 | -0.710769231 | -0.052808677 | -0.464270215 |
| R PRO314 | -0.251076923 | 0.110269231 | 0.359 | -0.001933754 | 0.216258554 |
| R GLU315 | -0.267576923 | -0.365692308 | 1.072153846 | -0.012184062 | 0.426700554 |
| R TYR316 | -0.111423077 | -0.073846154 | 0.101576923 | 0 | -0.083692308 |
| R LEU317 | -1.291307692 | 0.106076923 | -0.005807692 | -0.301572277 | -1.492610738 |
| R ALA318 | -0.074961538 | 0.063615385 | -0.052461538 | 0 | -0.063807692 |
| R PRO319 | -0.023653846 | 0.032269231 | 0.008961538 | 0 | 0.017576923 |
| R GLU320 | -0.055653846 | -0.251653846 | 0.169115385 | 0 | -0.138192308 |
| R VAL321 | -0.996076923 | 0.186576923 | -0.119846154 | -0.117979754 | -1.047325908 |
| R LEU322 | -0.1385 | 0.087115385 | 0.028846154 | -0.000743815 | -0.023282277 |
| R GLU323 | -0.031230769 | 0.144076923 | -0.040346154 | 0 | 0.0725 |
| R ASP324 | -0.055846154 | 0.198038462 | -0.086230769 | 0 | 0.055961538 |
| R ASN325 | -1.252 | 0.046307692 | 0.96 | -0.264190431 | -0.509882738 |
| R ASP326 | -0.125384615 | 0.0125 | 0.121307692 | 0 | 0.008423077 |
| R TYR327 | -2.755230769 | -0.171653846 | 1.959 | -0.379629692 | -1.347514308 |
| R GLY328 | -0.043769231 | -0.075346154 | 0.186230769 | 0 | 0.067115385 |
| R ARG329 | -0.019961538 | 0.198653846 | -0.170346154 | 0 | 0.008346154 |
| R ALA330 | -0.014 | -0.016884615 | -0.025153846 | 0 | -0.056038462 |
| R VAL331 | -0.197692308 | 0.012038462 | -0.098769231 | -0.009742985 | -0.294166062 |
| R ASP332 | -0.077846154 | -1.326269231 | 1.610769231 | 0 | 0.206653846 |
| R TRP333 | -0.016076923 | -0.010807692 | 0.026615385 | 0 | -0.000269231 |
| R TRP334 | -0.048230769 | 0.061692308 | -0.052692308 | 0 | -0.039230769 |
| R GLY335 | -0.0285 | 0.049538462 | -0.047076923 | 0 | -0.026038462 |
| R LEU336 | -0.014230769 | -0.000307692 | 0.007846154 | 0 | -0.006692308 |
| R GLY337 | -0.004384615 | 0.014807692 | -0.008115385 | 0 | 0.002307692 |
| R VAL338 | -0.026384615 | 0.032884615 | -0.060538462 | 0 | -0.054038462 |
| R VAL339 | -0.020538462 | 0.035615385 | -0.041384615 | 0 | -0.026307692 |
| R MET340 | -0.004076923 | 0.009153846 | 0.009038462 | 0 | 0.014115385 |
| R TYR341 | -0.004115385 | 0.020769231 | -0.017384615 | 0 | -0.000730769 |
| R GLU342 | -0.011038462 | -0.624307692 | 0.698230769 | 0 | 0.062884615 |
| R MET343 | -0.003230769 | 0.021923077 | -0.010961538 | 0 | 0.007730769 |
| R MET344 | -0.001115385 | 0.012461538 | -0.008615385 | 0 | 0.002730769 |
| R CYS345 | -0.000884615 | 0.019192308 | -0.013769231 | 0 | 0.004538462 |
| R GLY346 | -0.000807692 | 0.008076923 | 0.000346154 | 0 | 0.007615385 |
| R ARG347 | -0.002730769 | 0.196153846 | -0.174961538 | 0 | 0.018461538 |
| R LEU348 | -0.016961538 | 0.028307692 | -0.023230769 | 0 | -0.011884615 |
| R PRO349 | -0.004615385 | 0.017807692 | -0.004115385 | 0 | 0.009076923 |
| R PHE350 | -0.004884615 | 0.009269231 | 0.001807692 | 0 | 0.006192308 |
| R TYR351 | -0.004230769 | 0.0125 | -0.000076923 | 0 | 0.008192308 |
| R ASN352 | -0.000884615 | 0.002923077 | 0.001192308 | 0 | 0.003230769 |
| R GLN353 | -0.001884615 | -0.015884615 | 0.029307692 | 0 | 0.011538462 |
| R ASP354 | -0.002461538 | -0.072230769 | 0.095269231 | 0 | 0.020576923 |
| R HIE355 | -0.013192308 | -0.008576923 | 0.036 | 0 | 0.014230769 |
| R GLU356 | -0.007961538 | -0.029615385 | 0.098038462 | 0 | 0.060461538 |
| R ARG357 | -0.004538462 | 0.032961538 | -0.014076923 | 0 | 0.014346154 |
| R LEU358 | -0.0185 | 0.003576923 | -0.006038462 | 0 | -0.020961538 |
| R PHE359 | -0.024038462 | 0.001076923 | 0.027192308 | 0 | 0.004230769 |
| R GLU360 | -0.004 | -0.001538462 | 0.017038462 | 0 | 0.0115 |
| R LEU361 | -0.003769231 | 0.003115385 | 0.0125 | 0 | 0.011846154 |
| R ILE362 | -0.016038462 | -0.000653846 | 0.017615385 | 0 | 0.000923077 |
| R LEU363 | -0.008038462 | -0.007153846 | 0.015653846 | 0 | 0.000461538 |
| R MET364 | -0.001538462 | 0.000961538 | 0.002653846 | 0 | 0.002076923 |
| R GLU365 | -0.001307692 | -0.095230769 | 0.100884615 | 0 | 0.004346154 |
| R GLU366 | -0.000807692 | -0.111230769 | 0.116269231 | 0 | 0.004230769 |
| R ILE367 | -0.002 | 0.009153846 | -0.006461538 | 0 | 0.000692308 |
| R ARG368 | -0.000846154 | 0.098346154 | -0.095538462 | 0 | 0.001961538 |
| R PHE369 | -0.000961538 | 0.008923077 | -0.009192308 | 0 | -0.001230769 |
| R PRO370 | -0.000192308 | -0.000692308 | 0.000269231 | 0 | -0.000615385 |
| R ARG371 | -0.000269231 | 0.103807692 | -0.103423077 | 0 | 0.000115385 |
| R THR372 | -0.000115385 | 0.001961538 | -0.001923077 | 0 | -0.000076923 |
| R LEU373 | -0.000423077 | 0.008423077 | -0.007538462 | 0 | 0.000461538 |
| R SER374 | -0.000192308 | -0.006423077 | 0.006076923 | 0 | -0.000538462 |
| R PRO375 | -0.000076923 | -0.005961538 | 0.005692308 | 0 | -0.000346154 |
| R GLU376 | -0.000346154 | -0.152461538 | 0.151769231 | 0 | -0.001038462 |
| R ALA377 | -0.000346154 | -0.009076923 | 0.009076923 | 0 | -0.000346154 |
| R LYS378 | -0.000538462 | 0.138538462 | -0.138538462 | 0 | -0.000538462 |
| R SER379 | -0.000230769 | -0.007923077 | 0.008653846 | 0 | 0.0005 |
| R LEU380 | -0.001 | -0.005423077 | 0.007961538 | 0 | 0.001538462 |
| R LEU381 | -0.0015 | -0.011423077 | 0.012038462 | 0 | -0.000884615 |
| R ALA382 | -0.000730769 | -0.004423077 | 0.007576923 | 0 | 0.002423077 |
| R GLY383 | -0.000576923 | -0.000653846 | 0.001807692 | 0 | 0.000576923 |
| R LEU384 | -0.003961538 | -0.008384615 | 0.011230769 | 0 | -0.001115385 |
| R LEU385 | -0.005115385 | 0.009 | 0.005461538 | 0 | 0.009346154 |
| R LYS386 | -0.002692308 | 0.136615385 | -0.125615385 | 0 | 0.008307692 |
| R LYS387 | -0.004115385 | 0.078923077 | -0.047653846 | 0 | 0.027153846 |
| R ASP388 | -0.002423077 | -0.115 | 0.119615385 | 0 | 0.002192308 |
| R PRO389 | -0.006307692 | -0.016192308 | 0.031730769 | 0 | 0.009230769 |
| R LYS390 | -0.0025 | 0.044153846 | -0.025076923 | 0 | 0.016576923 |
| R GLN391 | -0.001192308 | -0.005461538 | 0.017153846 | 0 | 0.0105 |
| R ARG392 | -0.010576923 | 0.360846154 | -0.283 | 0 | 0.067269231 |
| R LEU393 | -0.001769231 | -0.022384615 | 0.026730769 | 0 | 0.002576923 |
| R GLY394 | -0.001423077 | -0.000730769 | 0.012653846 | 0 | 0.0105 |
| R GLY395 | -0.002115385 | 0.008615385 | 0.006730769 | 0 | 0.013230769 |
| R GLY396 | -0.000961538 | -0.014038462 | 0.017961538 | 0 | 0.002961538 |
| R PRO397 | -0.001038462 | -0.002576923 | 0.001461538 | 0 | -0.002153846 |
| R SER398 | -0.000423077 | -0.001307692 | 0.002769231 | 0 | 0.001038462 |
| R ASP399 | -0.000961538 | -0.185 | 0.196076923 | 0 | 0.010115385 |
| R ALA400 | -0.000961538 | 0.002153846 | -0.000307692 | 0 | 0.000884615 |
| R LYS401 | -0.0005 | 0.172576923 | -0.169961538 | 0 | 0.002115385 |
| R GLU402 | -0.000538462 | -0.167115385 | 0.167807692 | 0 | 0.000153846 |
| R VAL403 | -0.001038462 | 0.009884615 | -0.005769231 | 0 | 0.003076923 |
| R MET404 | -0.0005 | 0.005307692 | -0.0045 | 0 | 0.000307692 |
| R GLU405 | -0.000307692 | -0.149269231 | 0.148807692 | 0 | -0.000769231 |
| R HIE406 | -0.0005 | -0.003576923 | 0.003307692 | 0 | -0.000769231 |
| R ARG407 | -0.000153846 | 0.135615385 | -0.134615385 | 0 | 0.000846154 |
| R PHE408 | -0.000307692 | 0.004576923 | -0.005461538 | 0 | -0.001192308 |
| R PHE409 | -0.0005 | 0.002769231 | -0.005230769 | 0 | -0.002961538 |
| R LEU410 | -0.000115385 | 0.000384615 | -0.0005 | 0 | -0.000230769 |
| R SER411 | -0.000192308 | 0.000115385 | -0.000115385 | 0 | -0.000192308 |
| R ILE412 | -0.000346154 | 0.004346154 | -0.003807692 | 0 | 0.000192308 |
| R ASN413 | -0.000153846 | -0.007923077 | 0.008076923 | 0 | 2.732856676 |
| R TRP414 | -0.000653846 | -0.008692308 | 0.006884615 | 0 | -0.002461538 |
| R GLN415 | -0.000192308 | -0.005346154 | 0.005615385 | 0 | 0.000076923 |
| R ASP416 | -0.000115385 | -0.153346154 | 0.153153846 | 0 | -0.000307692 |
| R VAL417 | -0.000192308 | -0.000923077 | 0.002769231 | 0 | 0.001653846 |
| R VAL418 | -0.000153846 | -0.004346154 | 0.004807692 | 0 | 0.000307692 |
| R GLN419 | -0.000269231 | 0.003346154 | -0.003038462 | 0 | 0.000038462 |
| R LYS420 | -0.000269231 | 0.280846154 | -0.269692308 | 0 | 0.010884615 |
| R LYS421 | -0.000076923 | 0.137961538 | -0.137730769 | 0 | 0.000153846 |
| R LEU422 | -0.000115385 | -0.003307692 | 0.003538462 | 0 | 0.000115385 |
| R LEU423 | -0.000230769 | -0.003461538 | 0.003846154 | 0 | 0.000153846 |
| R PRO424 | -0.000269231 | 0.0105 | -0.010153846 | 0 | 0.000076923 |
| R PRO425 | -0.000346154 | 0.007576923 | -0.007884615 | 0 | -0.000653846 |
| R PHE426 | -0.000423077 | -0.005807692 | 0.005692308 | 0 | -0.000538462 |
| R LYS427 | -0.000076923 | 0.156115385 | -0.154192308 | 0 | 0.001846154 |
| R PRO428 | -0.000269231 | 0.005461538 | -0.005615385 | 0 | -0.000423077 |
| R GLN429 | -0.000192308 | 0.003307692 | -0.003076923 | 0 | 0.000038462 |
| R VAL430 | -0.000230769 | 0.000153846 | 0.000576923 | 0 | 0.0005 |
| R THR431 | -0.000038462 | 0.000576923 | -0.000423077 | 0 | 0.000115385 |
| R SER432 | -0.000115385 | -0.003346154 | 0.003576923 | 0 | 0.000115385 |
| R GLU433 | -0.000346154 | -0.130230769 | 0.130269231 | 0 | -0.000307692 |
| R VAL434 | -0.000115385 | 0.001076923 | -0.001076923 | 0 | -0.000115385 |
| R ASP435 | -0.000038462 | -0.119115385 | 0.118807692 | 0 | -0.000346154 |
| R THR436 | -0.000346154 | 0.003 | -0.001923077 | 0 | 0.000730769 |
| R ARG437 | -0.000269231 | 0.115192308 | -0.113076923 | 0 | 0.001846154 |
| R TYR438 | -0.001038462 | 0.002884615 | 0.000461538 | 0 | 0.002307692 |
| R PHE439 | -0.001615385 | 0.004807692 | -0.005769231 | 0 | -0.002576923 |
| R ASP440 | -0.0005 | -0.085923077 | 0.087846154 | 0 | 0.001423077 |
| R ASP441 | -0.000576923 | -0.108807692 | 0.114769231 | 0 | 0.005384615 |
